# Synergetic anticancer activity of *psidium guajava*–mediated palladium nanoparticles via apoptosis induction and metastasis suppression in osteosarcoma cells

**DOI:** 10.1038/s41598-026-49913-1

**Published:** 2026-05-10

**Authors:** Demiana H. Hanna, Basma M. Taher, Mohamed A. El-Desouky

**Affiliations:** 1 Department of Chemistry, Faculty of Science, Cairo University, Giza, 12613 Egypt; 2 Faculty of Science, Cairo University National University, Giza, Egypt

**Keywords:** Biosynthesized palladium nanoparticles, MG-63 bone cancer cells, Apoptosis, Cell cycle arrest, ELISA technique, Biochemistry, Biotechnology, Cancer, Drug discovery, Nanoscience and technology

## Abstract

Bone cancer is an uncommon type of malignant tumor that begins in bone tissue and can destroy normal bone structure or develop as metastases from another malignancy. At 30% of all bone cancer cases, osteosarcoma is the most typical primary malignant bone cancer. It frequently appears in the metaphyses of adolescent long bones, which have the most capacity for growth. Therefore, the recent study aims to synthesize safer phytoconstituent mediated- palladium nanoparticles (PdNPs) utilizing aqueous leaves extract of *Psidium guajava* (PGLE) and for insight into the produced nanoparticles’ therapeutic impacts on the pathways that inhibit MG-63 cancer cell lines. The obtained PdNPs displayed amorphous spherical morphology with an average diameter of 4.72±1.616  nm and a zeta potential value of -14.8  mV. The extract-suspended palladium nanoparticles (PdNPs-PGLE) exhibited a lower IC_50_ value of (89.81 ± 0.32 µg/ml) and had a superior cytotoxic effect towards MG-63 cell growth compared to the PGLE (110.65 ± 1.07µg/ml) and PdNPs (198.22 ± 0.24µg/ml) samples. It was shown that (PdNPs-PGLE) increased significantly the % of total apoptotic cells compared to PGLE and PdNPs with higher intensity comet nucleus in treated cells compared to control cells. It is shown that there was an arrest in the S cell cycle phase in MG-63-treated cells. The PdNPs-PGLE had a significant decrease in wound closure % (45.21) compared to control untreated cells (58.78). Furthermore, the PdNPs-PGLE severely suppresses the capacity of MG-63 of colony formation, with a plating efficiency of 0.9% and a surviving fraction of 0.009, compared to control cells. Additionally, the estimation of the expression of protein levels using the ELISA technique revealed that there was an increase in the Bax, cleaved active Caspase-3, and TP53 expression levels with a decrease in the Bcl-2, CDK2, MMP-2, and MMP-9 expression in the PdNPs-PGLE-treated cells compared to control cells *(p* < 0.05) .These findings point out the potential of *Psidium guajava*–mediated green synthesis not only as an eco-friendly route for nanoparticle fabrication but also as a means to harness phytochemical–nanomaterial synergy.

## Introduction

Cancer is a complex disease characterized by excessive cell proliferation, resistance to apoptosis, ongoing angiogenesis, and the ability to invade and metastasis to distant organs. These hallmarks result from genetic abnormalities and epigenetic changes that impair essential regulatory processes controlling cell cycle progression, DNA repair, and cellular homeostasis^1,2^. Osteosarcoma (OS) is one of the most common primary malignant bone tumors, particularly in children and adolescents^3^.It is distinguished by aggressive growth and a high propensity for metastasis, particularly to the lungs. Osteosarcoma is more likely to arise in the metaphysis of long bones, particularly the distal femur (43%), proximal tibia (23%), and humerus (10%). Despite breakthroughs in treatment, survival rates remain low due to chemoresistance and tumor recurrence^4^.

The current treatment strategy for osteosarcoma is multimodal, involving surgery and systemic chemotherapy. Surgical resection remains the cornerstone of therapy, with the goal of completely removing the primary tumor with acceptable margins, but limb-salvage techniques are now recommended over amputation wherever possible. This is usually paired with neoadjuvant and adjuvant chemotherapy, such as high-dose methotrexate, doxorubicin, and cisplatin (MAP treatment), which has considerably improved survival rates in patients with localized illness^5^. However, treatment-related toxicity, chemoresistance, and poor outcomes in recurrent or metastatic cases continue to pose significant clinical problems.

Nanotechnology is the design of materials at the nanoscale, where they have unique physicochemical properties that differ from those of bulk materials^6^. Nanotechnology has enabled the development of nanomedicine, which provides potential tools for cancer detection, targeted therapy, and theragnostic applications^7,8^. These nanosystems may improve therapy efficacy and reduce off-target toxicity. Due to the potential pharmaceutical and biological uses of metallic nanoparticles (MNPs), there is increasing demand for non-toxic, environmentally friendly, and economical production techniques^9^.

Metallic nanoparticles have emerged as viable therapeutic agents in osteosarcoma treatment due to their distinct physicochemical qualities, which include a large surface area, variable size, and the possibility to be functionalized with bioactive compounds. These nanoparticles, including gold, silver, palladium, and iron oxide, can cause cytotoxicity in osteosarcoma cells by a variety of methods. One of the principal impacts is the production of reactive oxygen species (ROS), which causes oxidative stress, mitochondrial malfunction, DNA damage, and, eventually, apoptosis. Furthermore, metallic nanoparticles can disrupt cell cycle progression and affect important signaling pathways involved in tumor growth and survival, such as the PI3K/Akt and MAPK pathways. Their tiny size also allows for improved cellular absorption and preferential accumulation in tumor tissues through the enhanced permeability and retention (EPR) effect^10–13^.

A growing amount of research is focused on creating nanoscale metals using physical, chemical, and green synthesis techniques^14^. Higher expenses, radiation exposure, high temperature, energy, and pressure requirements, poorer thermal stability, a lot of waste, high dilution, challenging shape and size tunability, and a decreased likelihood of stability are the disadvantages of physical methods of synthesis^15^, and the main drawback of the chemical synthesis of nanoparticles is the use of organic solvents and harsh reducing agents such as sodium borohydrideand sodium citrate. These chemical reagents provide environmental and toxicological risks^16^.

Due to worries about high energy consumption, dangerous chemical emissions, the requirement for specialized equipment, and synthesis conditions, physical and chemical.

procedures are gradually being replaced by green synthesis techniques^14^. Green synthesis of nanoparticles uses plants or plant parts to reduce metal ions into their elemental form in the size range of 1 to 100 nm^17^, and biological synthesis utilizes fungi, algae, and bacteria to create nanoparticles. In addition to being more cost-effective, efficient, and straightforward, the green synthesis technique is also easily scalable to carry out larger operations^16^. These extracts contained biomolecules like proteins, polysaccharides, enzymes, and polyphenols that formed nanoparticles from reduced metal ions^18^. The three most important conditions for NPs’ green synthesis are the selection of a safe stabilizing agent, an acceptable non-toxic reducing agent, and green- friendly solvent like water, ethanol, and their mixes^7^.

Palladium nanoparticles (PdNPs) are gaining popularity due to their unique physicochemical properties, which include tunable size and morphology, a high surface-to-volume ratio, and remarkable chemical stability, making them promising candidates for a variety of applications^19,20^. In addition to their well-known roles in catalysis and sensing, PdNPs have shown promise in biomedical sectors due to their antioxidant, antibacterial, and anticancer properties, as well as their minimal toxicity to normal cells^19,21^. Despite these promising characteristics, the biological application of PdNPs has received less attention than its industrial and catalytic uses, emphasizing the necessity for more research into their therapeutic potential^22^.

The guava tree, or *Psidium guajava*, is widely distributed in Africa, Asia, Eastern Europe, and other continents. It is grown for its juicy fruits, therapeutic qualities, and aesthetic appeal. It is a member of the Magnoliophyta phylum, Magnoliopsida class, and Myrtaceae family. Particularly in guava leaves, a wide range of bioactive substances are included, such as phenolics, flavonoids, tannins, minerals, essential oils, ascorbic acid, proteins, and polysaccharides^23^. The leaves, fruits, bark, and roots of the *Psidium guajava* plant have long been used as a topical remedy or as an oral decoction, infusion, and cooked preparation to cure a variety of illnesses. These compounds were used to treat rheumatism, diabetes mellitus, obesity, hypertension, and gastrointestinal disorders like stomachaches and diarrhea because of their analgesic and antibacterial qualities. The high concentration of carotenoids, essential oils, flavonoids, phenolic compounds, and vitamins in the leaves and fruits of the *Psidium guajava* plant is responsible for their therapeutic qualities^24^. *Psidium guajava* leaves extract participated in the green synthesis of many types of nanoparticles, like silver nanoparticles^25^, iron oxide nanoparticles^26^, zero-valent iron nanoparticles^27^, titanium dioxide nanoparticles^28^, gold nanoparticles^29^, and copper oxide nanoparticles^30^.

Although various research has been conducted to examine plant-mediated palladium nanoparticle production and anticancer efficacy, most of them have focused solely on palladium-purified nanoparticles. The biosynthesis of palladium nanoparticles from an aqueous extract of *Psidium guajava* without the use of any effects^31^, the efficacy of the prepared palladium nanoparticles as an anticancer agent against MG-63, and the comparative evaluation of extract-suspended palladium nanoparticles and their corresponding purified forms were all limited. Quantitative assessment of biological efficacy, including statistical analysis of factors like IC_50_, had not been adequately addressed. As a result, our study seeks to fill this gap by rigorously assessing the anticancer efficacy of both systems under identical experimental conditions. So, the present study aims to green synthesize palladium nanoparticles by using aqueous *psidium guajava* leaves extract and to determine the chemical characteristics for the biogenic palladium nanoparticles such as shape, size, stability, and crystallinity. Moreover, to examine the therapeutic impacts of the extract-suspended palladium NPs on the mechanisms of inhibition of human MG-63 cancer cells using an MTT assay, Annexin V/PI staining assay, Comet assay, cell cycle assay, wound healing assay, clonogenic assays, and ELISA techniques.

## Materials and methods

### Chemicals and reagents

Palladium (II) chloride (anhydrous 60% basis) was purchased from Sigma-Aldrich, Nasr City, Egypt. All additional chemical reagents used were readily available and of commercial quality.

## Methods

### Preparation of palladium chloride solution

The palladium (II) chloride solution (5 mM) was prepared first as described before^32^. In brief, palladium (II) chloride (5 mM) was added to warmed distilled water (60 °C) and placed in the magnetic stirrer until it completely dissolved.

Preparation of aqueous *Psidium guajava* leaves extract (PGLE).

The *Psidium guajava* leaves (Voucher No. m-332) were collected from the local market. The aqueous *Psidium guajava* leaves extract (PGLE) was prepared as described before^32^. About 3 g of dried *Psidium guajava* leaves were washed with deionized water and dissolved in 100 ml of distilled water. Then, the prepared leaves were boiled for 30 min at 60 °C. After that, the obtained extract was cooled, filtered, and reserved at 4 °C for further investigations.

## Determination of phytochemical compounds

### Total phenolic content determination

As previously described^33^, the Folin-Ciocalteu reagent was used to determine the total phenolic content of the aqueous *Psidium guajava* leaves extract (PGLE) using a colorimetric approach. To prepare the solution, 1 ml of the extract was mixed with  2 ml of methanol, and then 500 µL portions of this extract were mixed with 2.5 mL of sodium carbonate (75 g/L) and a tenfold diluted Folin-Ciocalteu reagent. After mixing for ten seconds using a vortex, the tubes were allowed to sit at 25 °C for a duration of two hours. The absorbance was then measured at 765 nm, using the reagent blank as a reference. Gallic acid equivalent (GAE) milligrams per gram were used to express the total phenolic content^34^. The following formula was then used to calculate the total amount of phenolics in the PGLE extract using the GAE calibration curve:

TPC = C x V/M.

where V is the volume of the (PGLE) extract solution in milliliters, M is the mass of the (PGLE) extract in grams, and C is the concentration of GAE in milligrams per milliliter that was determined using the calibration curve. Total phenolic content, or TPC, is measured in mg GAE/g of the extract (PGLE)^35^.

### Total flavonoid content determination

The total flavonoid content for the PGLE was implemented by following the steps of an earlier report^36^, according to the modified AlCl₃ colorimetric method. In short, two milliliters of methanol was mixed with one milliliter of extract . After adding 200 µl of extract to 75 µl of 5% NaNO_2_, the mixture was allowed to sit at room temperature for five minutes. Each vial was then filled with 0.5 ml of NaOH and 1.25 ml of AlCl₃. After that, it was sonicated and let to sit at room temperature for five minutes. Following incubation, a methanol blank at 510 nm was used to measure the absorbance of all working solutions and standard solutions^34^. The quercetin standard calibration curve was used to determine the extract’s flavonoid content. The results were then reported as micrograms of quercetin equivalent (QE) per 1 g of dry extract using the coming formula:

C=(c x v)/m.

where C is the total amount of flavonoid components expressed as mg of QE/g of the PGLE extract, V is the extract’s volume (measured in ml), m is the weight of the tested extract (measured in g), and c is the amount of QE obtained using the calibration curve (measured in mg/ml)^35^.

### Synthesis of palladium nanoparticles

**Fig. 1:**
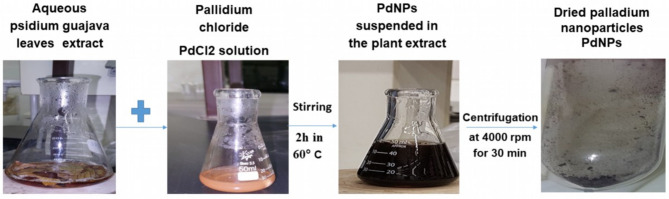
The steps of synthesis palladium nanoparticles (PdNPs) from the aqueous *psidium guajava* leaves extract (PGLE).

The palladium nanoparticles were prepared according to the previous study^32^. The steps of the synthesis of palladium nanoparticles were displayed in Fig. 1. For the preparation of the palladium nanoparticles, the palladium chloride solution was added to the warmed PGLE, and the mixture was placed in a magnetic stirrer for 2 h at 60 °C. Then, the obtained biogenic palladium nanoparticle solution was centrifuged, washed, and preserved for further investigation.

## Characterizations of the obtained palladium nanoparticles

### UV-Visible spectroscopy

UV-visible spectroscopy was used for confirming the preparation of the biogenic palladium nanoparticles and for the determination of their optical properties. The optical analysis was performed for aqueous *Psidium guajava* leaves extract, the palladium chloride solution, and the dried palladium nanoparticles using a Shimadzu UV-1800 UV/visible scanning spectrophotometer at the wavelength range 200–800 nm^37^.

### X-ray diffraction analysis (XRD)

The Bruker D8 Discover instrument was used to measure the crystallite size and crystalline structure of biogenic dried palladium nanoparticles. A (2θ) value between 10° and 80° was used to record the diffractogram^32^.

### Transmission Electron microscopy (TEM)

The size distribution and shape of the green-produced dried palladium nanoparticles (PdNPs) were determined at different magnifications using the JEM-2100 electron microscope^38,39^.

### Fourier Transform Infrared Spectroscopy (FTIR)

The FTIR spectra were executed using Jasco-4100 for both the aqueous *psidium guajava* leaves extract and dried palladium nanoparticles to determine the existence of different functional groups in the aqueous *psidium guajava* leaves extract that act as reducing agents^40,41^.

### Scanning Electron Microscopy (SEM)

Using a Tescan SEM (TESCAN VEGA 3, Czech Republic), scanning electron microscopy examination was used to assess the shape of the palladium nanoparticles. A Quorum Technologies Ltd. sputter coater (Q150t, England) was used to coat the dried palladium nanoparticles with gold (Au) for 120 s after they had been adhered to aluminum microscope stubs using adhesive carbon tape^42,43^.

### Zeta potential measurement

Zeta potential was employed to assess the stability of the palladium nanoparticles (PdNPs) and of extract-suspended palladium nanoparticles (PdNPs-PGLE) using Malvern Instruments Ltd^44^.

### Biological activity

#### Cell line preparation

The American Type Culture Collection (ATCC) supplied the MG-63 cell line (which was derived from Homo sapiens, humans, and possessed fibroblast morphology and was extracted from the bone) and the WI-38 cell line (which was derived from Homo sapiens, humans, and possessed fibroblast morphology and was extracted from the lung). They were cultivated in base Eagle’s Minimum Essential Medium with heat-inactivated fetal bovine serum up to a final concentration of 10% for MG-63 and with fetal bovine serum up to a final concentration of 10% for WI-38 and were exposed to an environment that included 95% air and 5% CO₂ at 37 °C.

### The MTT assay

The MTT assay is a colorimetric *in vitro* method employed to quantify the proportion of metabolically active MG-63 cancer cells and cytotoxic activity for the prepared aqueous *Psidium guajava* leaves extract (PGLE), palladium nanoparticles (PdNPs), and the extract- suspended palladium nanoparticles (PdNPs-PGLE) as previously reported^45,46,47]^. In brief, 1 × 10^5^ cells/ml (100 µL/well) were injected onto 96-well tissue culture plates, which were then cultivated for 24 h at 37 °C. The cell monolayer was then given two washes. The RPMI medium (maintenance medium) containing 2% serum was used to dilute the tested samples twice. In separate wells, 100 µl of each dilution were assessed. Each tested substance was administered to the MG-63 cells at varying doses (31.25–1000 µg/ml). The plate was examined after being incubated at 37 °C. Any external signs of toxicity, such as rounding, shrinkage, cell granulation, or partial or complete loss of the monolayer, were looked for in the cells. To completely incorporate MTT into the media, each well was then given twenty microliters of MTT solutions (5 mg/mL in PBS) and shaken for five minutes at 150 rpm. In order to metabolize MTT, the cells were exposed to 5% CO_2_ for one to five hours at 37 °C. To completely dissolve the formazan in the solvent, it was placed on a shaking table set at 150 rpm for five minutes. Under a microscope, the optical density was determined at 560 nm^48^. Using the following formulas, the impact of the tested sample concentrations on the growth of MG63 was expressed as a percentage of cytotoxicity^49^.

$$\:Cell\:viability\:\left(\%\right)=\frac{\mathrm{A}\mathrm{v}\mathrm{e}\mathrm{r}\mathrm{a}\mathrm{g}\mathrm{e}\:\mathrm{o}\mathrm{f}\:\mathrm{t}\mathrm{h}\mathrm{e}\:\mathrm{a}\mathrm{b}\mathrm{s}\mathrm{o}\mathrm{r}\mathrm{b}\mathrm{a}\mathrm{n}\mathrm{c}\mathrm{e}\:\mathrm{o}\mathrm{f}\:\mathrm{t}\mathrm{h}\mathrm{e}\:\mathrm{t}\mathrm{r}\mathrm{e}\mathrm{a}\mathrm{t}\mathrm{e}\mathrm{d}\:\mathrm{c}\mathrm{e}\mathrm{l}\mathrm{l}\mathrm{s}\:}{\mathrm{A}\mathrm{v}\mathrm{e}\mathrm{r}\mathrm{a}\mathrm{g}\mathrm{e}\:\mathrm{o}\mathrm{f}\:\mathrm{t}\mathrm{h}\mathrm{e}\:\mathrm{a}\mathrm{b}\mathrm{s}\mathrm{o}\mathrm{r}\mathrm{b}\mathrm{a}\mathrm{n}\mathrm{c}\mathrm{e}\:\mathrm{o}\mathrm{f}\:\mathrm{t}\mathrm{h}\mathrm{e}\:\mathrm{u}\mathrm{n}\mathrm{t}\mathrm{r}\mathrm{e}\mathrm{a}\mathrm{t}\mathrm{e}\mathrm{d}\:\mathrm{c}\mathrm{e}\mathrm{l}\mathrm{l}\mathrm{s}}X\:100\:Cytotoxicity\:\left(\%\right)=100-Viability\left(\%\right)$$

The 50% inhibitory concentration (IC_50_) of PGLE, PdNPs, and PdNPs-PGLE was determined following a four-hour incubation period at 37 °C. The IC_50_ concentration is the concentration at which half of the cells remain viable. To evaluate the biocompatibility of the extract-suspended palladium nanoparticles on healthy tissues, the entire MTT experiment was conducted against healthy embryonic lung cells (WI-38) at concentrations ranging from 31 to 1000 µg/ml.

### Annexin V/PI staining assay

The annexin V/PI staining assay was used to determine the percentage of apoptosis and necrosis of the osteosarcoma cell line (MG-63) that was treated with each PGLE, PdNPs, and PdNPs-PGLE separately using the Annexin V-FITC Apoptosis Detection Kit (catalog: K101-25) according to the previous method^50^. The phosphatidylserine (PS) that translocated to the outer cell membrane following the start of the apoptotic process was highly attractive to Annexin V-fluorescein isothiocyanate (FITC)^51^. To put it briefly, after being harvested, the cells were put on a 6-well culture plate with 1 × 10^6^ cells per well, and they were left to incubate for the entire night. The MG-63 cells were then exposed to each of the three treatments’ IC_50_ values for 48 h. Following trypsinization, centrifugation, and PBS rinsing, the treated cells were collected and resuspended in 500 µl of binding buffer (1X). Additionally, the cells were incubated for five minutes at room temperature in the dark after 5 µl of Annexin V-FITC and 5 µl of propidium iodide (50 mg/ml) were added. Lastly, Annexin V-FITC binding (excitation maximum = 488 nm; emission maximum = 530 nm) was examined using flow cytometry with the FITC signal detector and PI staining using the phycoerythrin emission signal detector.

### Cell cycle assay

The cellular DNA content in the MG-63 cells that were treated with PGLE, PdNPs, and PdNPs-PGLE was detected using the Propidium Iodide Flow Cytometry Kit (Catalog ab139418, Abcam) according to the previous method^52^. Concisely, the MG-63 cells were cultured in a 6-well culture plate with 1 × 10^6^ cells per well and left to let cell adhesion occur. Then, the cells were incubated with the IC_50_ values of the three treatments for 48 h and stored at 4 °C for 4 weeks. After the cells had been trypsinized, centrifuged, and rinsed with PBS, the treated cells were collected and resuspended in 200 µL of Propidium Iodide (1X) in addition to RNase Staining Solution. After 20 to 30 min of dark incubation at 37 °C, the cells were ready for flow cytometry analysis. Lastly, the intensity of propidium iodide fluorescence was measured using a flow cytometer’s FL2 and a 488 nm laser excitation [excitation maximum = 493 nm; emission maximum = 636 nm].

### Clonogenic assay

The clonogenic assay, an *in vitro* technique for assessing cell viability, is employed to evaluate the ability of a single cell to proliferate and form a colony^53^. The determination of the effects of PdNPs-PGLE on MG-63 cells was performed as previously mentioned^54^. Briefly, after being placed on a 6-well plate, the cells were incubated at 37 °C and 5% CO_2_ for a full day. During the incubation period, the cells were treated with PdNPs-PGLE at an IC_50_ concentration. The incubated cells were subsequently incubated for a duration of one to three weeks or until colony formation was observed. Following cell fixation with 6% glutaraldehyde, crystal violet (0.5%) staining was applied for 30 min. Following a tap of wash, the cells were left to dry at room temperature. After the cells had dried, a stereomicroscope was utilized to see the colonies, as well as a colony counter pen to count them automatically. Finally, equations were used to calculate plating efficiency (a measure of colony production capability) and surviving fraction (SF), respectively.

% Plating efficiency (PE) = Number of former colonies/Number of plated cells X 100.

Surviving fraction (SF) = PE of treated sample/PE of control.

#### Cell scratch wound healing assay

The invasion of target tissues by malignant cells results in the formation of numerous tumors. Therefore, one of the strategies to stop cancer from spreading is to obstruct the adhesion, motility, and invasion of target organs by cancer cells. So, a scratch wound healing assay was performed to study the effect of the PdNPs-PGLE on the migration of osteosarcoma cells (MG-63)^55,56^. In brief, cells were cultured at 37 °C on a 6-well plate until 90% of them were confluent as a monolayer. Then, to simulate a wound, a pipette tip was used to perform an artificial straight scratch. A light microscope was used to take images of the created scratch, and it was subsequently washed three times using phosphate buffer saline. The scratched cells were seeded in fresh media containing the tested PdNPs-PGLE, and the untreated cells were seeded in media containing sterile saline. The cells were then cultured for 48 h at 37 °C with 5% CO₂. The migration of treated and untreated cells was evaluated again after 48 h of incubation. Finally, using equations, the rate of cell migration and percentage of wound closure were calculated according to scratched cells’ width at 0 and 48 h.

Rate of cell migration (RM) = (wi – wf)/t.

Wi =average of the initial width of the wound (µm).

Wf= average of final width of the wound (µm).

t = span time of the assay in (hours).

Percentage of wound Clouser (%) = {(At = 0 – At=∆t)/At = 0} 100.

At = 0 = initial wound area.

At=∆t = wound area after n hours.

#### Comet assay

The comet assay is a widely used technique for evaluating DNA fragmentation in apoptotic cells. This method involves the formation of a comet tail, which occurs when cellular DNA is fragmented and separated from intact DNA under the influence of an electrophoretic field^57,58^. In a nutshell, PdNPs-PGLE was applied to MG-63 cells for 48 h. Then, 75 µl of low melting point agarose was added to the cell suspension and placed into the base slides that were prepared previously from 1% to 0.5% low melting point agarose (LMPA) and 1.0% normal melting point (NMA) and then placed into lysing solution for 2 h at 4 °C. After removing the slides from the lysing solution, the slides were put into the horizontal gel box, which was filled with the alkaline buffer for 20 min. The slides were then electrophoresed for 30 min and then let sit in neutralization buffer three times for 5 min. The slides were initially treated with 80 µL of ethidium bromide (1X) for a duration of 5 min. Subsequently, the DNA stained with EtBr was observed using a 40x objective lens on a fluorescent microscope. The resulting images were analyzed with Komet 5 image analysis software, developed by Kinetic Imaging, Ltd. (Liverpool, UK), which was connected to a CCD camera. This analysis aimed to evaluate both the quantitative and qualitative aspects of DNA damage in the cells by measuring the length of DNA migration and the percentage of DNA that had migrated. Ultimately, the software calculated the tail moment. Typically, 50 to 100 cells were randomly selected for analysis in each sample.

#### ELISA testing

The expression levels of the proteins involved in apoptosis (Bax, Bcl-2, TP53, and cleaved caspase-3), cell cycle regulation (CCNA and CDK2), and metastasis (MMP-9 and MMP-2) in the extract-suspended palladium nanoparticles (PdNPs-PGLE)-treated and untreated MG-63 cells were evaluated and quantified using the ELISA technique, and optical density was quantified using the ROBONIK P2000 ELISA reader. The used kits were the human Bax ELISA kit (EIA-4487) that was provided by DRG International, Inc. (USA); the human Bcl-2 ELISA kit (99 − 0042) that was provided by Invitrogen Corporation (Carlsbad, USA); the human Active Caspase-3 (KHO1091) that was provided by Invitrogen Corporation (Camarillo, USA); the human TP53 (Tumor protein P53)ELISA kit (E-EL-H0910) that was provided by Elabscience; the human CDK2 ELISA kit (LS-F27663) that was provided by LifeSpan BioSciences, Inc.; the human CCNA ELISA kit (MBS8804448) that was provided by MyBioSource; the human MMP-9 simple step ELISA kit (ab246539); and the human MMP-2 ELISA kit (ab100606) that was provided by Abcam.

### Antioxidant activity using DPPH Assay

The antioxidant potential of palladium nanoparticles suspended in extract (PdNPs-PGLE) was evaluated through the DPPH radical scavenging assay. The ability of PdNPs-PGLE to neutralize DPPH radicals was determined following established protocols^59,60^. Specifically, the free radical scavenging activity was assessed using 1,1-diphenyl-2-picrylhydrazyl (DPPH). A 0.1 mM DPPH solution was prepared in ethanol, to which 1 ml was combined with 3 ml of the sample dissolved in ethanol at varying concentrations (3.9, 7.8, 15.62, 31.25, 62.5, 125, 250, 500, and 1000 µg/ml), with ascorbic acid serving as the reference standard. The mixtures were vigorously agitated and incubated at ambient temperature for 30 min. Subsequently, absorbance was recorded at 517 nm using a UV-Vis spectrophotometer (Milton Roy). The half-maximal inhibitory concentration (IC_50_), representing the concentration required to inhibit 50% of the DPPH radicals, was derived from the logarithmic dose-response inhibition curve. The percentage of DPPH radical scavenging activity was calculated according to the equation:

DPPH scavenging effect (%) or Percent inhibition = A0 – A1/A0 × 100.

Where A0 was the absorbance of the control reaction and A1 was the absorbance in the presence of the test or standard sample.

### Statistical analysis

Statistical analyses were performed using SPSS Statistics software. Comparisons between two groups were conducted using an independent samples t-test. For experiments involving more than two groups, one-way analysis of variance (ANOVA) followed by Tukey’s multiple comparisons post hoc test was applied to assess differences among group means. Data are presented as mean ± SD from at least three independent experiments, and a *p* value < 0.05 was considered statistically significant.

## Results and Discussion

### Determination of the total phenolic and flavonoids content

The secondary plant metabolites found in *Psidium guajava*, especially its leaves, contain specific polyphenols that may have anti-inflammatory, inherent antiviral, and antioxidant qualities. A number of guava components that have been most commonly reported, like ascorbic acid (vitamin C), lycopene, and flavonoids (apigenin), have anticancer effects *in vitro*^61^. The composition of *Psidium guajava* contains phenolic compounds, particularly flavonoids, which are potent antioxidants that are crucial in blocking the body’s production of free radicals. As a result, they can help prevent cancer and premature skin aging^61^. The obtained findings of the total phenolic and total flavonoids in the aqueous *Psidium guajava* leaves extract (PGLE) were 18.85 ± 0.14 mg (GAE)/ml and 21.91 ± 0.12 mg (QuE)/ml, respectively. The amounts of the phenolics and flavonoids found in the aqueous *Psidium guajava* leaves extract may have effects as antioxidant agents in the reduction of the reaction of palladium nanoparticle formation. According to previous reports, the identification of *Psidium guajava* leaf extracts revealed that the plant is an abundant source of a phenolic compound that may be crucial for the bio-reduction of metal ions (M+) to metal nanoparticles (Mo). The phenolic substances taken out of *Psidium guajava* leaves help reduce Ag + and stabilize Ag + to AgNPs, because these phenolic compounds can donate electrons^62^.

### UV-visible spectroscopy

**Fig. 2:**
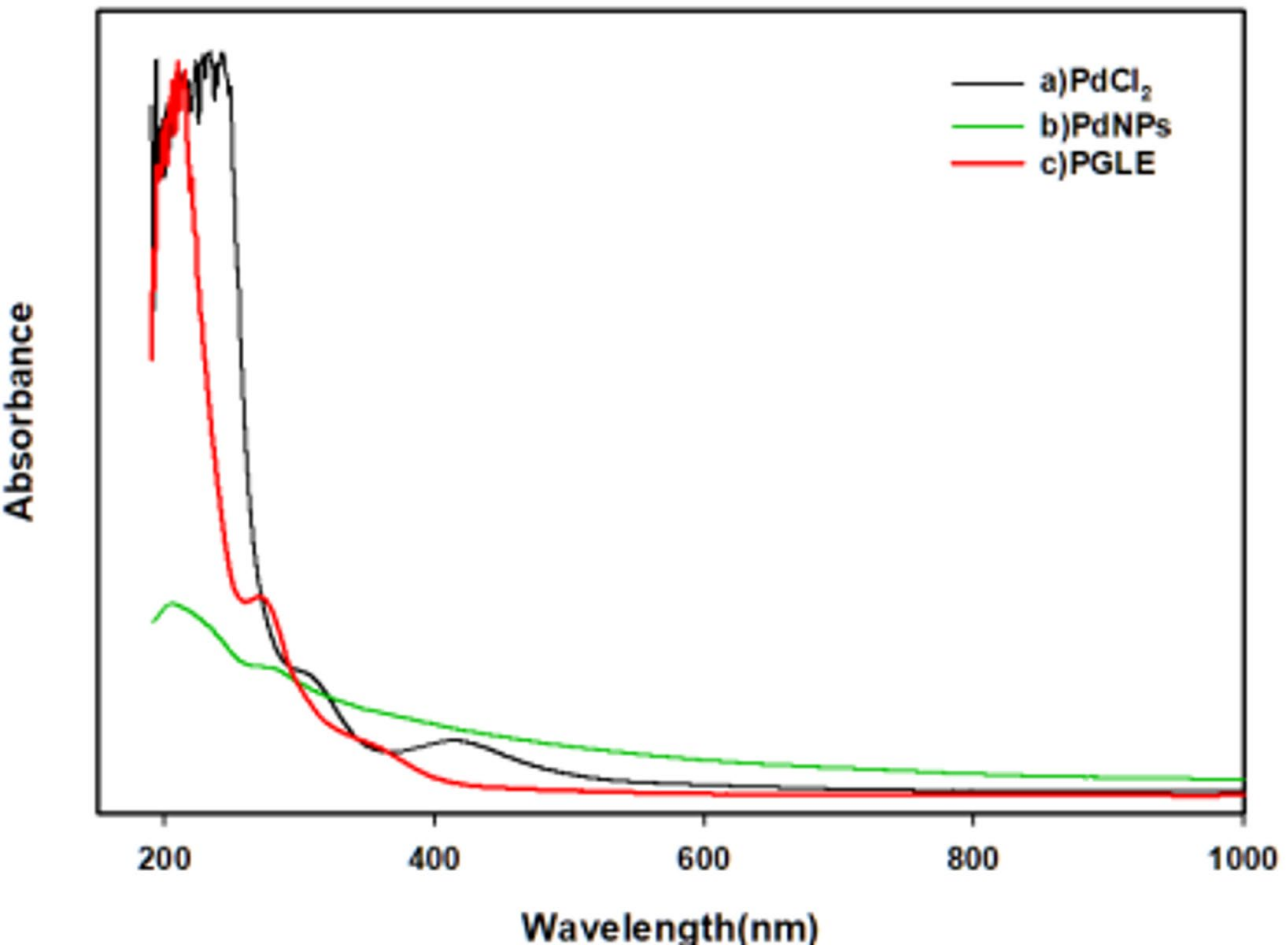
UV-Visible Spectroscopy images of (a) Palladium chloride (PdCl_2_), (b) Palladium nanoparticles (PdNPs), and (c) Aqueous *psidium guajava* leaves extract (PGLE).

In this study, an aqueous extract of *Psidium guajava* leaves was employed as a reducing agent for palladium chloride (PdCl₂). The formation of palladium nanoparticles (PdNPs) was monitored through UV–visible spectrophotometry. The progressive color transition from a colorless solution to a black colloidal suspension served as confirmation of the successful synthesis of PdNPs. The UV spectrum of each of the aqueous *Psidium guajava* leaves extract, palladium chloride, and dried palladium nanoparticles was shown in Fig. 2. The UV spectrum of the aqueous *Psidium guajava* leaves extract (PGLE) had an absorption band at 269 nm. The UV spectra related to the palladium chloride (PdCl₂) showed a broad continuous absorption band at 415 nm and a very intense band at 242 nm. In the dried palladium nanoparticles (PdNPs) spectrum, there was an absorption band at 206 nm and a disappearance of the 415 nm absorption band. Similarly, a previous study showed that the dH₂O extract of the *Psidium guajava (guava)* leaf extract revealed absorbance peaks at 214 nm, 268 nm, and 350 nm^37^. Also, the absorption peak at 268 nm was recorded in an earlier study of aqueous extract of leaves of *Psidium guajava*^63^. Moreover, the presence of a 415 nm absorption peak may be due to palladium ions (Pd²⁺) existing in the palladium chloride spectrum, and the disappearance of this peak in the palladium nanoparticle spectrum was agreed with by the study of palladium nanoparticle formation using the crude extract of Sargassum bovinum reported in earlier research^64^. Thus, the palladium nanoparticle formation was due to the reduction of the palladium ions (Pd²⁺) to (Pd⁰) as a result of the phenolic compounds’ existence.

#### X-ray diffraction analysis

**Fig. 3:**
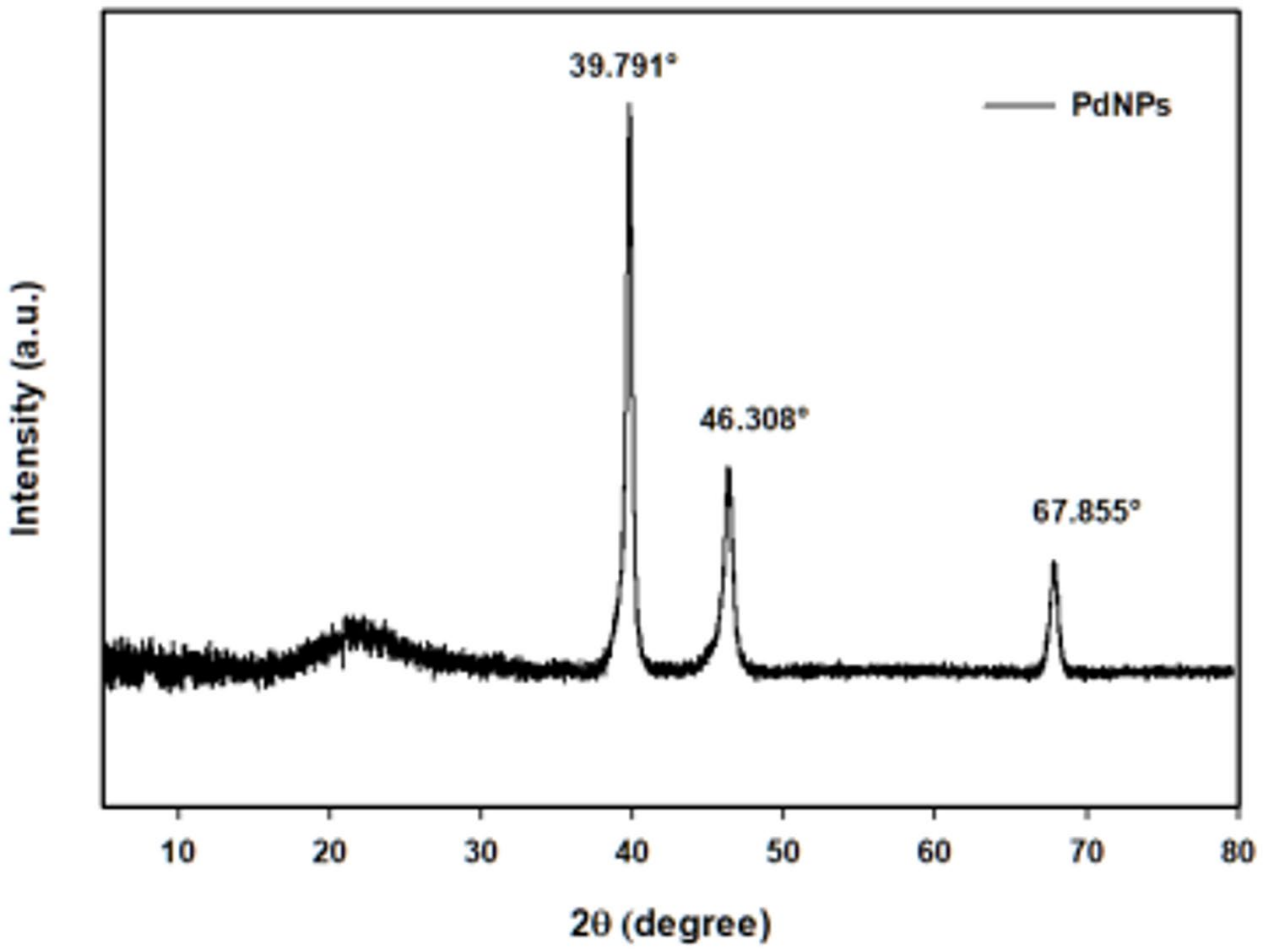
XRD figure for PdNPs synthesized from the aqueous *Psidium guajava* leave extract.

The obtained diffractogram of palladium nanoparticles (PdNPs) that are synthesized by aqueous *Psidium guajava* leaves extract is represented in Fig. 3. As shown in Fig. 3, there were three distinct diffraction peaks, which are located at the following positions: 39.791°, 46.308°, and 67.855°, corresponding to the following lattice planes: (111), (200), and (220), respectively. The crystallinity of the phyto-palladium nanoparticle corresponded to the Joint Committee on Powder Diffraction Standards (JCPDS). This XRD pattern indicated that the crystalline structure of the green synthesized palladium nanoparticles was a face-centered cubic crystal (fcc). The average crystalline size was calculated by the following equation of Debye-Scherrer, and the average crystalline size was 15.26 nm.

D = k λ l β cos θ.

Where D represents the average particle size, λ represents the wavelength of the X-ray source (λ = 1.54060°A), β is the full width of the half maxima (FWHM), K is the Scherrer coefficient, which equals 0.9, and θ is Bragg’s angle. Similarly, Narasaiah et al.. mentioned earlier that the XRD pattern of prepared Pd NPs from the extract of the *Pimpinella tirupatiensis* plant showed characteristic diffraction peaks at 39.550°, 46.220°, and 67.570°, which correspond to the (111), (200), and (220), respectively, with an average crystalline size equal to 15.4 nm^32^.

#### Transmission electron microscopy (TEM)

**Fig. 4:**
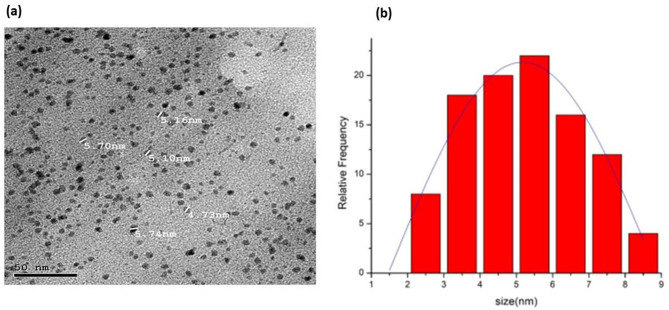
Transmission Electron Microscope (TEM) image for the biogenic palladium nanoparticles synthesized by *Psidium guajava* leave extract (a), and its respective particle size distribution histogram (b).

TEM analysis reveals that the average particle size of the Phyto synthesized palladium nanoparticles was 4.72 ± 1.616 nm, as displayed in Fig. 4(a, b). Similarly, the previously produced palladium nanoparticles using the Cinnamomum camphora leaf broth had a size range from 3.0 to 5.0 nm^38^. Because of their higher surface area to volume ratio, nanoparticles have a greater surface area of contact per mass unit than larger particles, causing some normally inert compounds, such as gold, to become reactive in the nanometer range^65^. Nanoparticles, due to their small, controlled size, can rapidly permeate body tissues and fluids that would be impossible to reach in bulk form^65^. In essence, the size and surface area of these particles determine their rate of endocytosis, distribution, retention, and elimination in biological systems^66^.

## Fourier transform infrared spectroscopy

**Fig. 5:**
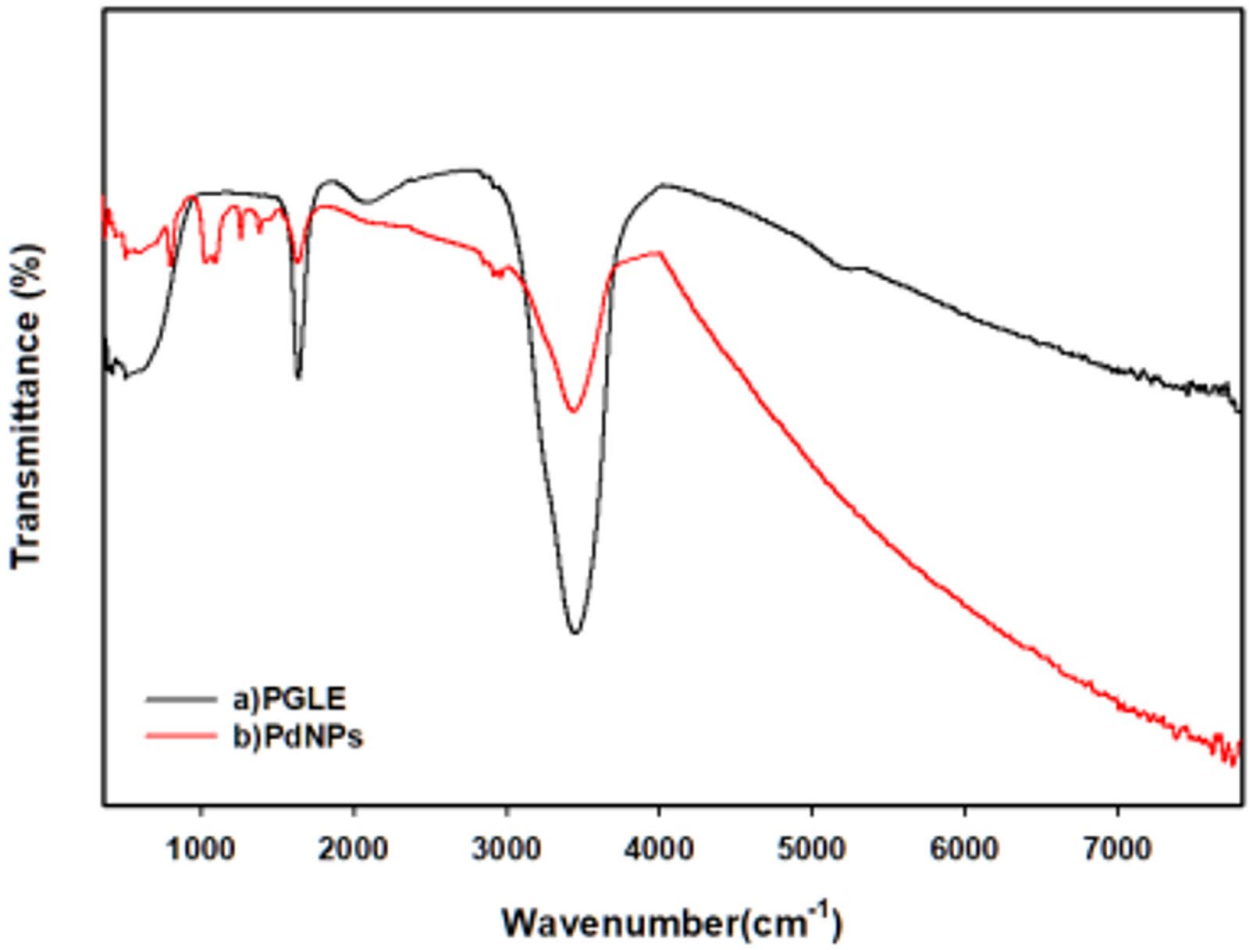
The FTIR spectrum for (a) aqueous *Psidium guajava* leaves extract (PGLE), and (b) dried palladium nanoparticles (PdNPs).

The FTIR spectra of the aqueous *Psidium guajava* leave extract (PGLE) were represented in Fig. 5a. The absorption bands in the spectrum of PGLE were observed at 3447.13 cm⁻¹, 2919.7 cm⁻¹, 2851.24 cm⁻¹, 2382.62 cm⁻¹, 2075.3 cm⁻¹, 1637.27 cm⁻¹, 1464.67 cm⁻¹, 1383.64 cm⁻¹, and 508.151 cm⁻¹. In the spectra of PGLE, there was a very intense broad absorption band at 3447.13 cm⁻¹ that corresponds to the hydrogen-bonded hydroxyl group (-OH) stretching frequency that is found in the phenolic compounds. The presence of 2919.7 cm⁻¹ and 2851.24 cm⁻¹ may represent the stretching vibration of the (C-H) bond in the CH₂ group for asymmetric and symmetric stretching, respectively^67^. The absorption band at 2382.62 cm⁻¹ is attributed to O-H stretching vibrations of carboxylic acid groups^68^.The absorption band at 2075.3 cm⁻¹ is attributed to the stretching vibration of alkyne (C ≡ C) functional groups present in the phytochemical constituents of the extract^69,70^. The existence of 1637.27 cm⁻¹ may be attributed to nonconjugated stretching vibration (-C = C), and the bending vibration of the germinal methyl group is represented by 1383.64 cm⁻¹^40^. The absorption band at 1464.67 cm⁻¹ corresponds to the antisymmetric deformation of CH₃ groups in aliphatic compounds^71^. The band at 508.151 cm⁻¹ was detected; however, its precise assignment could not be conclusively determined and may be related to skeletal vibrations of complex phytochemical constituents.

The FTIR spectra of dried palladium nanoparticles (PdNPs) were represented in Fig. 5b. In this spectrum, there were many bands similar to those that exist in the spectrum of aqueous *Psidium guajava* leave extract with very little shift. The transmittance located in the PdNps spectra was observed at 3434.6 cm⁻¹, 2963.09 cm⁻¹, 2919.7 cm⁻¹, 2852.2 cm⁻¹, 1633.41 cm⁻¹, 1383.68 cm⁻¹, 1261.22 cm⁻¹, 1093.44 cm⁻¹, 1029.8 cm⁻¹, and 802.242 cm⁻¹. The presence of wavenumber 3434.6 cm⁻¹ may be indicated by the presence of the hydrogen-bonded hydroxyl group (-OH) stretching frequency^41^. The absorption band at 2963.09 cm⁻¹ is attributed to C-H stretching vibrations of aliphatic hydrocarbon groups The appearance of this peak could be due to possible interactions of functional groups with metal ions during reduction and atoms or smaller NPs during capping^72^. The presence of 2919.7 and 2852.2 may represent the stretching vibration of the (C-H) bond of CH₂ and the presence of (C = C) or an aromatic ring may be represented by the existence of 1633.41 cm⁻¹^41^. The bending vibration of the germinal methyl group is represented by 1383.68 cm⁻¹^41^. The absorption bands at 1261.22 and 1093.44 cm⁻¹ are attributed to C-O stretching vibrations of alcohols, carboxylic acids, esters, and ether groups present in the phytochemical constituents capping the surface of PdNPs^73^. The absorption band at 1029.8 cm⁻¹ is attributed to C-O stretching vibrations of alcoholic groups, which have been reported to contribute to the reduction and stabilization of PdNPs^74^. The band at 802.24 cm⁻¹ may correspond to out-of-plane bending vibrations of aromatic C–H groups associated with phytochemical constituents adsorbed on the nanoparticle surface.

## Scanning electron microscopy

**Fig. 6:**
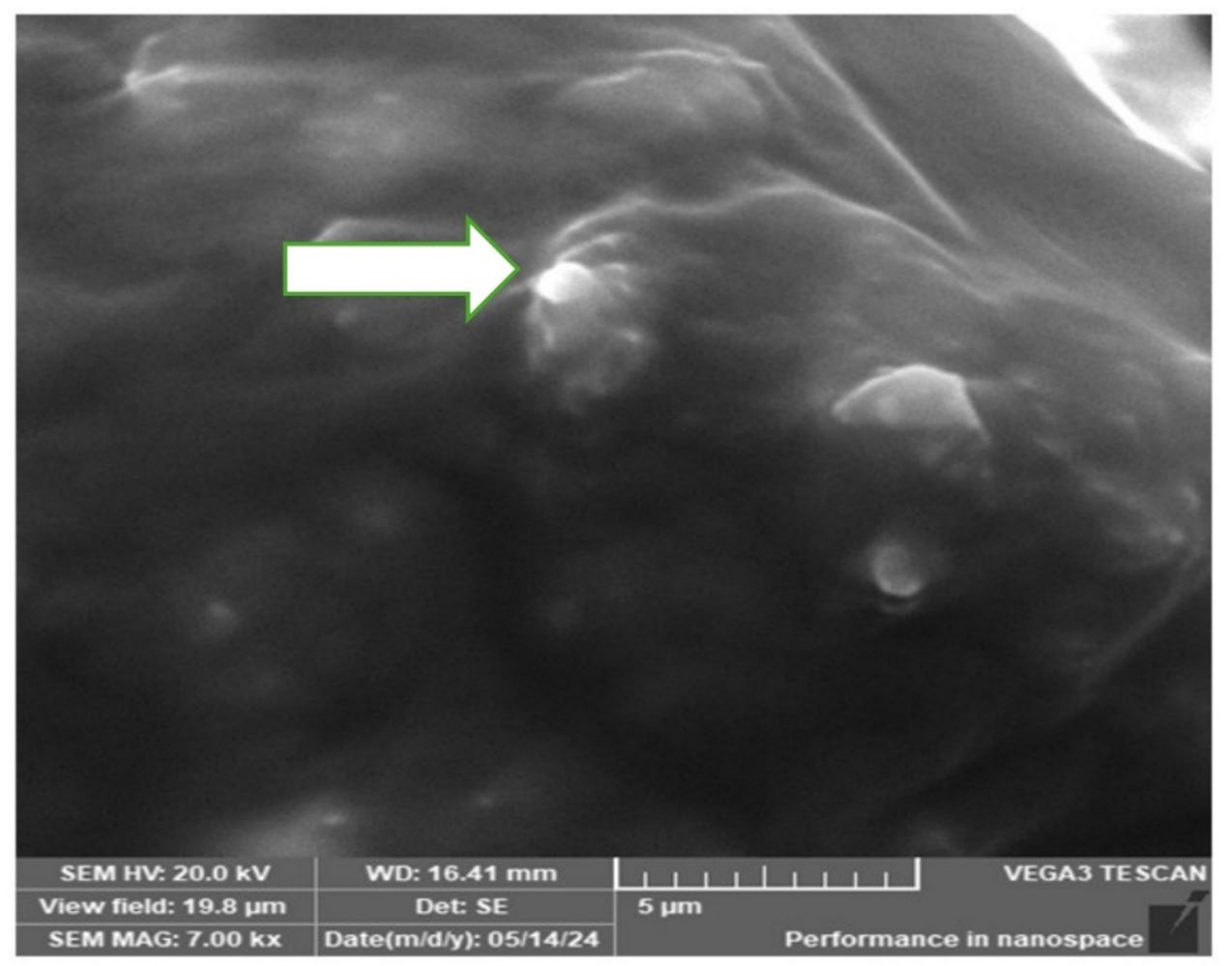
Scanning electron microscope (SEM) image of palladium nanoparticles (PdNPs) synthesized by *Psidium guajava* leave extract.

The morphology of the palladium nanoparticles that were detected by the scanning electron microscope was spherical (Fig. 6), as small spherical white spots, and this was close to the previous SEM image of the synthesized palladium nanoparticles formed by Hippophae rhamnoides *Linn leaf* extract^42^.

### Zeta potential measurement

**Fig. 7:**
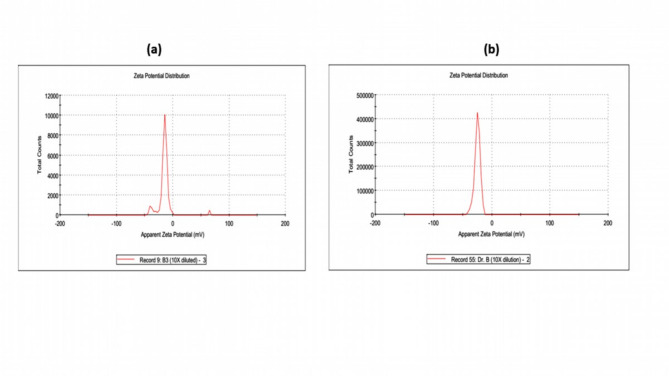
a) Zeta potential of Palladium nanoparticles PdNPs and (b) the extract- suspended Palladium nanoparticles (PdNPs-PGLE).

The observed zeta potential of the palladium nanoparticles (PdNPs) and the extract-suspended palladium nanoparticles (PdNPs-PGLE) is shown in Figs. 7a and b. The obtained zeta potential of PdNPs and PdNPs-PGLE was − 14.8 mV and − 24.9 mV, respectively. These results confirmed the stability of both PdNPs and PdNPs-PGLE and their abilities to be used in the biological applications related to the previous research that demonstrated that the stability of the nanoparticles that had zeta potential values in the range from + 30 to −30 mV avoided the aggregation of the nanoparticles and supplied enough repulsion forces^75,76^. The PdNPs-PGLE were more stable than palladium nanoparticles (PdNPs), which might be due to the phytocompounds in the plant extract appearing to be effectively capping, as indicated by the negative zeta potential, which results in electrostatic particles repelling one another, preventing agglomeration^77^.

#### MTT Assay

**Fig. 8:**
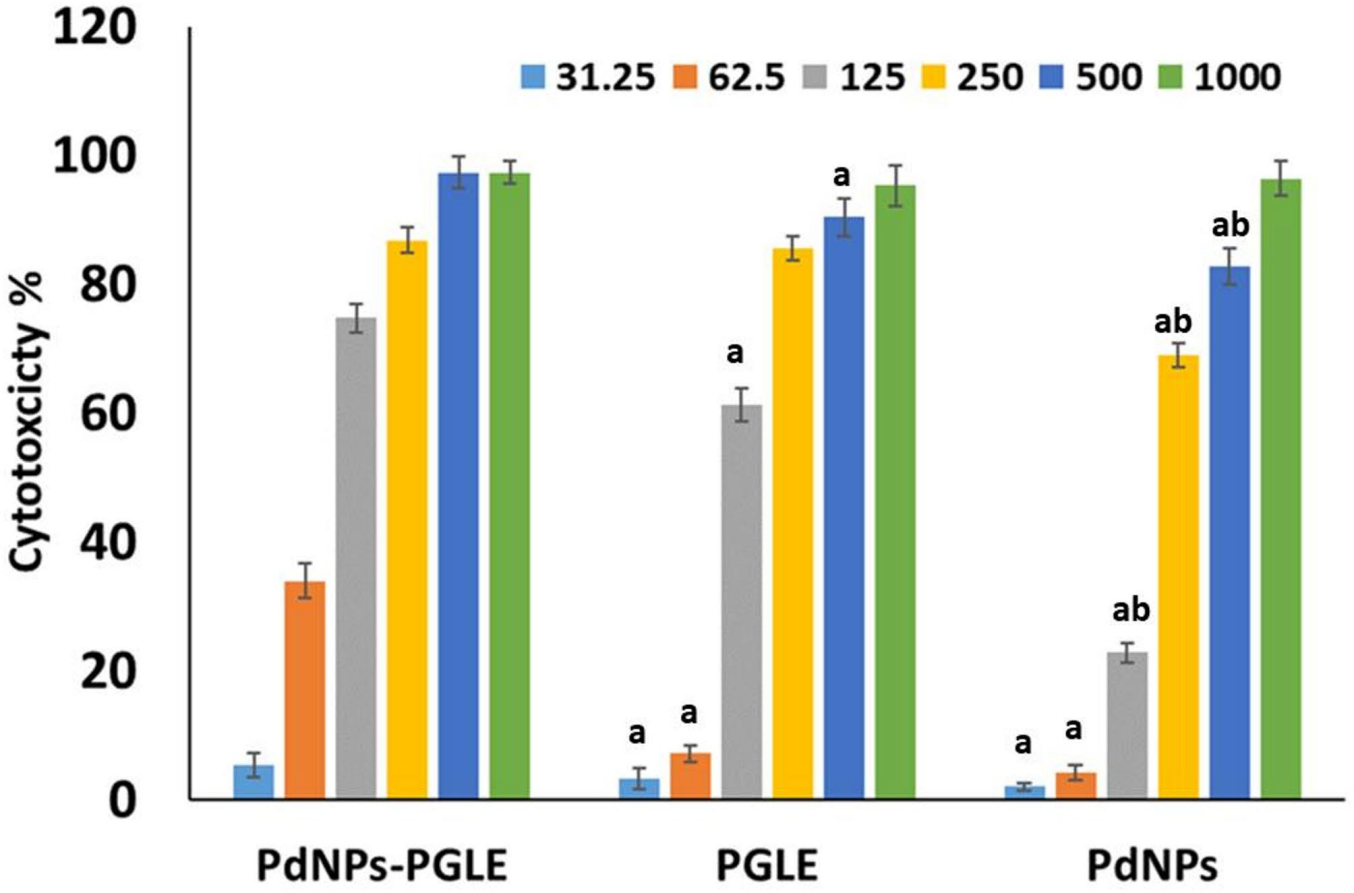
The cytotoxic effect of the samples PGLE, PdNPs and PdNPs-PGLE at various concentrations (31.25–1000 µg/ml) on MG-63 cancer cells using the MTT test, Data are presented as mean ± SD of three separate analyses. Statistically significant (*p* < 0.05) differences are shown by lowercase letters: (a) (*p* < 0.05) concerning the PdNPs-PGLE group, and b (*p* < 0.05) concerning PGLE group.

**Fig. 9:**
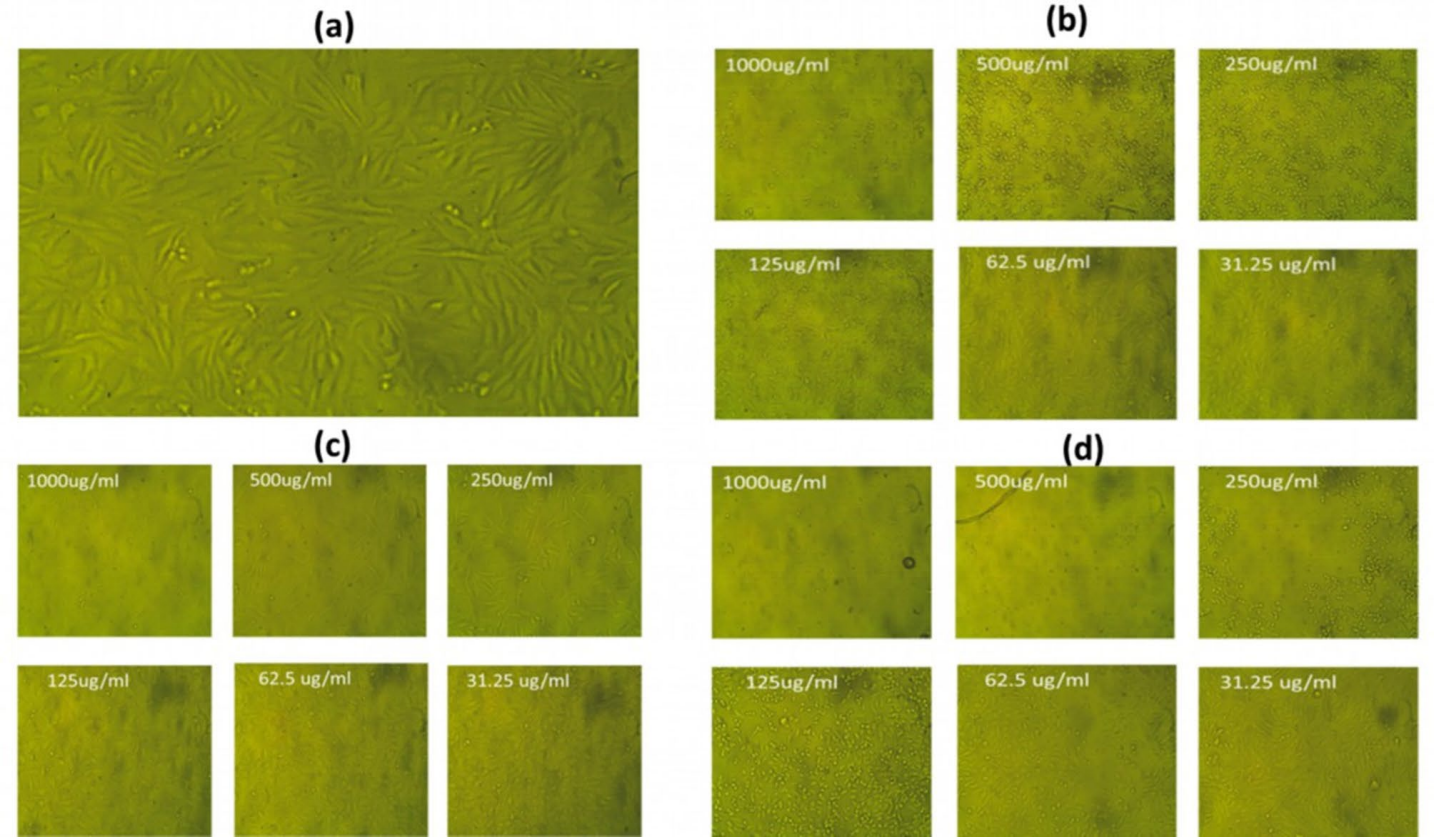
(b) The morphological changes in MG-63 cancer cells based on 24 h treatment with different dosages of the PGLE, (c) PdNPs and (d) PdNPs-PGLE samples compared to (a) normal control cells.

**Fig. 10:**
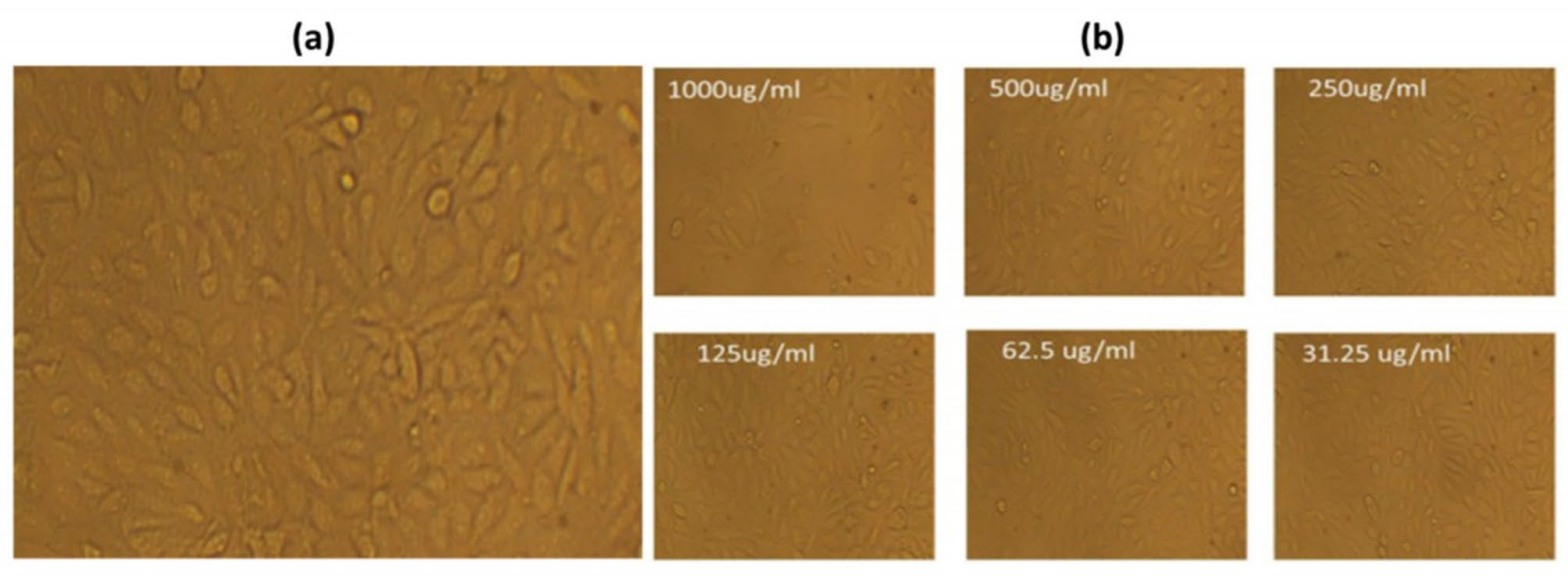
(b) The morphological changes in normal WI-38 cells based on 24 h treatment with different dosages of the PdNPs-PGLE sample in contrast to(a)control untreated MG-63 cells.

A comparative study was performed among different concentrations (31.25–1000 µg/ml) of each aqueous *Psidium guajava* leaf extract (PGLE), biogenic palladium nanoparticles (PdNPs), and extract-suspended palladium nanoparticles (PdNPs-PGLE) to study their cytotoxicity against MG-63 cells using the MTT assay for 24 h. It was found that the increase in the concentration of all tested samples (31.25–1000 µg/ml) through incubation for 24 h significantly increased the cytotoxicity percentage against the treated MG-63 cells as shown in Figure (8). It was shown from the obtained results that the PdNPs-PGLE had the highest percentage of cytotoxicity through the tested concentration with the strongest IC_50_ concentration (89.81 ± 0.32 µg/ml) compared to the PGLE (110.65 ± 1.07 µg/ml) and PdNPs (198.22 ± 0.24 µg/ml) samples. Additionally, the PdNPs-PGLE showed safety and biocompatibility characteristics against normal WI-38 cells with higher IC_50_ concentration (660.75 ± 2.76 µg/ml), where it showed higher cell viability % through all its tested concentrations. Cell morphological changes, such as granulation, vacuolization in the cell’s cytoplasm, and rounding or shrinking of the cells, can all be seen under a microscope and were considered indicators of cytotoxicity^78.^  As shown in Fig. 9 (a-d), in contrast to the control untreated MG-63 (Fig. 9a), which exhibited a robust adherent cell that grew on a monolayer of epithelial cells, the PGLE (Fig. 9b), PdNPs (Fig. 9c), and PdNPs-PGLE (Fig. 9 d), respectively, showed highly morphological apoptotic variations in MG-63 cells, such as cell shrinkage, phase-dense nucleus, rounded cell, and decreasing in the number of cells by increasing the tested samples’ concentration. While the PdNPs-PGLE sample didn’t show any morphological apoptotic variations in normal WI-38 cells (Fig. 10b) compared to control normal WI-38 cells (Fig. 10a).

Compared to *Psidium guajava* fruit, *Psidium guajava* leaves exhibited a greater capacity for antioxidants^79^ Gallic acid, chlorogenic acid, ferulic acid, caffeic acid, and ellagic acid were polyphenols, which were the main antioxidant components extracted from *Psidium guajava* leaves^79^. Vitamin C, flavonoids (kaempferol, myricetin, quercetin, epicatechin, catechin, and rutin), anthocyanins, tannins (ellagic acid and procyanidin B2), terpenoids (limonene and β-caryophyllene), and terpenoids (limonene) were well known for their anti-cancer and potent anti-inflammatory properties^79^, and many of these compounds were found in the aqueous *Psidium guajava* leaf extract according to the previous studies^80,81^. Therefore, the highest cytotoxic activity of the extract-suspended palladium nanoparticles (PdNPs-PGLE) against MG-63 might be due to the combination of both the anticancer activity of the flavonoid and phenolic components found in the PGLE that could inhibit cell proliferation and activate apoptosis and the anticancer activity of biogenic palladium nanoparticles (PdNPs). Furthermore, it was discovered that *Psidium guajava* leaf components, isolated quercetin fractions, and quercetin derivatives such as quercetin-3-O-xylopyranoside and quercetin-3-O-arabinopyranoside had an effect against oxidative stress caused by CCl₄ by preventing cellular proliferation and triggering apoptotic pathways. These pathways resulted in programmed cell death by disrupting the integrity of the mitochondrial membrane and increasing membrane permeability^79^.

Additionally, previous research by Gnanasekara et al.. showed that the fruit extract of *Couroupita guianensis Aubl.* (CGFE) was used for preparing the green synthesized palladium nanoparticles (CGPdNPs) with size ranges from 5 to 15 nm, where the IC_50_ values of CGFE and CGPdNPs against lung cancer cells (A549) were determined to be 160 and 121 µg/ml, respectively^45^. In another study, the green synthesized palladium nanoparticles using the extract of Saudi Propolis showed effective anticancer activity with an IC_50_ of 104.79 µg/mL against ductal carcinoma (MCF-7)^48^. Gulbagca et al. reported that the preparation of PdNPs from *Urtica plant* extract showed anticancer properties against the human pancreatic cancer cells (MIA PaCa-2), human colon cancer cells (HT-29), and human breast cancer cells (MDA-MB-231)^82^. In another previous study, the MTT assay results for PdNPs synthesized from the aqueous extract of Agaricus bisporus exhibited an excellent cytotoxic effect against the kidney cancer cell line (PK13)^75^.

### Annexin V/PI staining assay

**Fig. 11:**
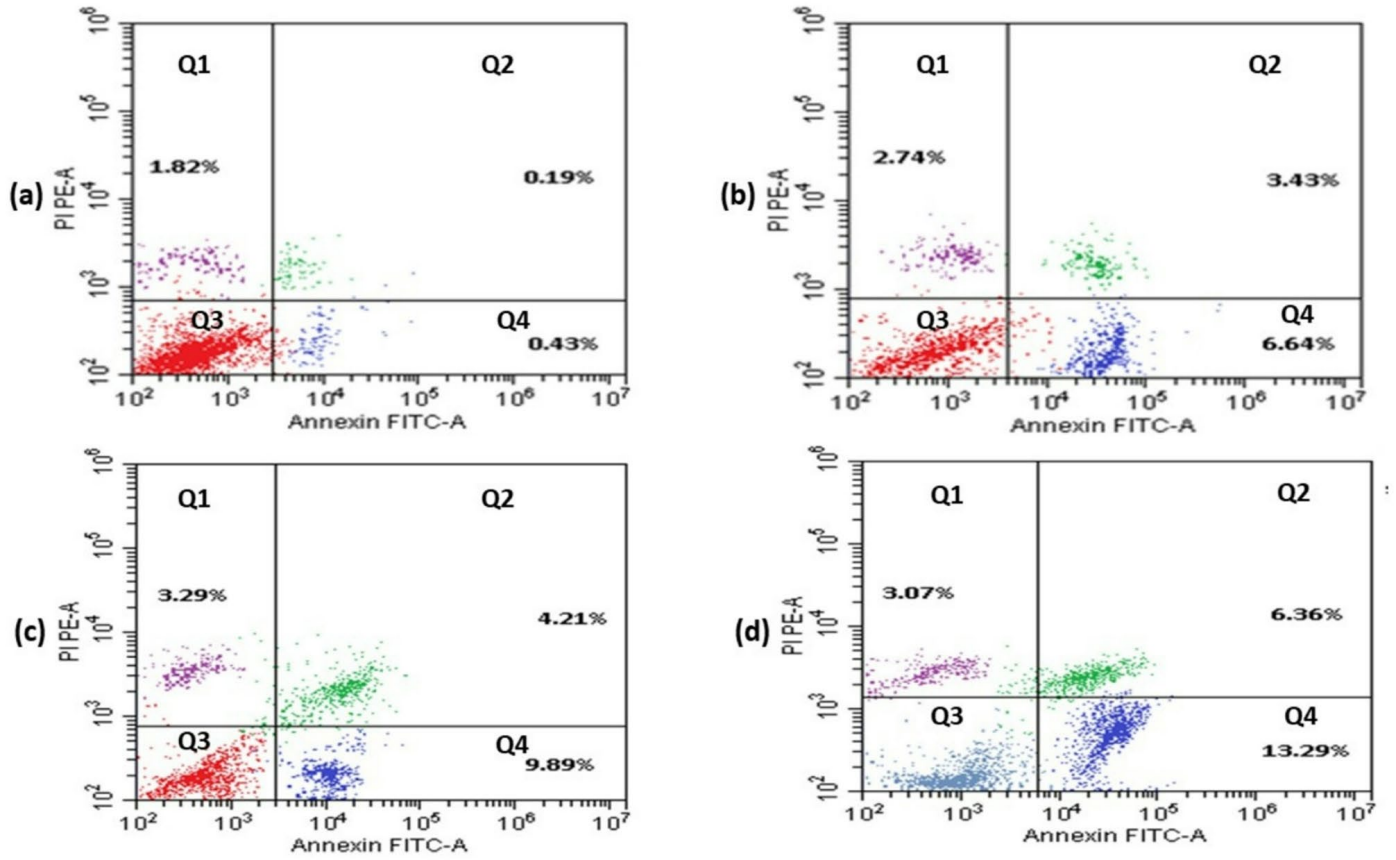
The apoptotic manner in MG-63 cells was measured by flow cytometry, which revealed Q1 (An−, PI+) percentage of necrotic cells, Q2 (An+, PI+) percentage of late apoptotic cells, Q3 (An−, PI−) percentage of viable cells, and Q4 (An+, PI−) percentage of early apoptotic cells for treated cells (b) with an IC_50_ dose of PGLE, (c) PdNPs, and (d) PdNPs-PGLE, in contrast to (a) untreated control MG-63 cells.

**Fig. 12:**
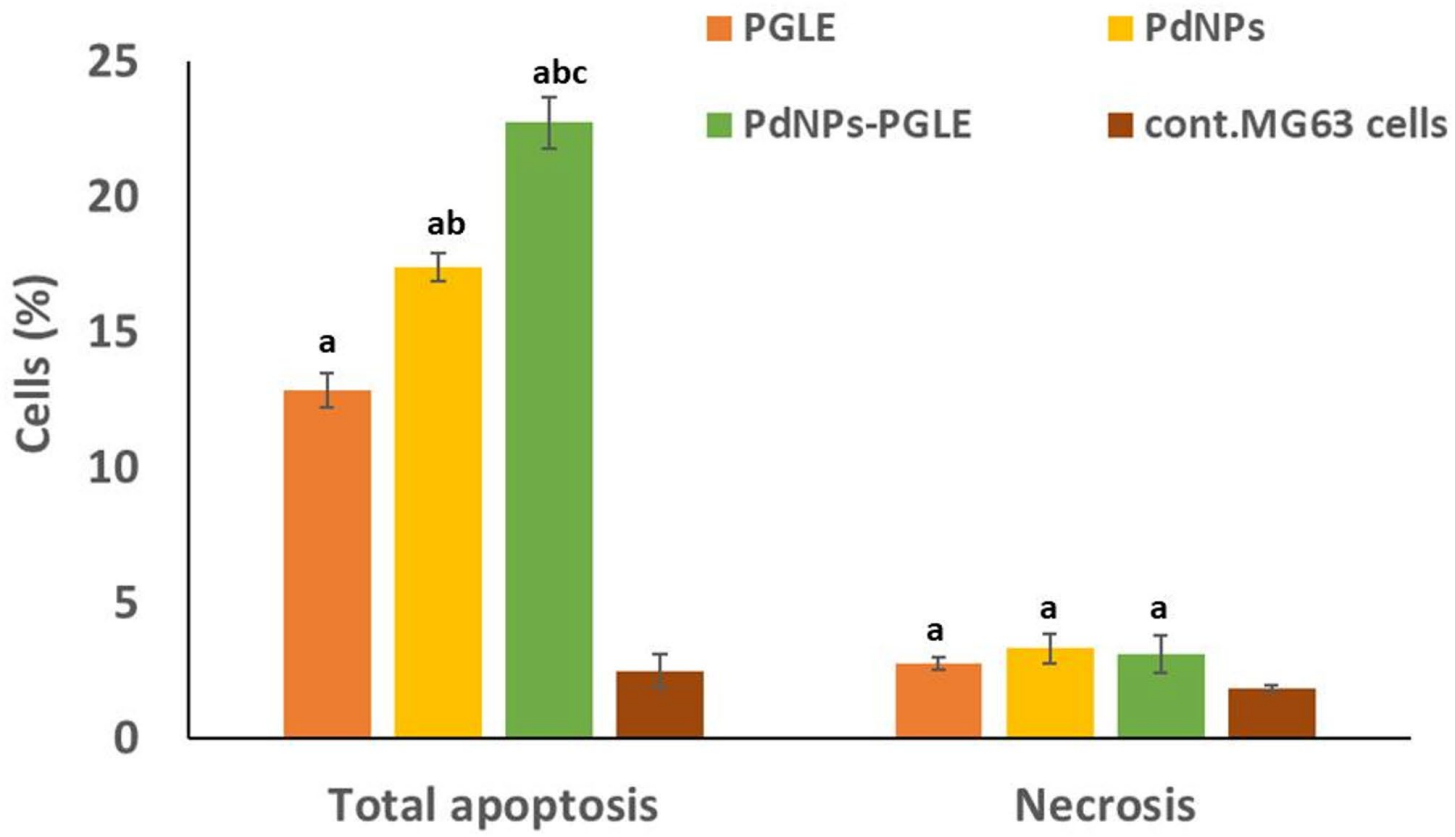
Comparative analysis of total apoptosis (early and late), and necrosis percentage in MG-63 cells treated with an IC_50_ dose of PGLE, PdNPs, and PdNPs-PGLE in contrast to untreated control MG-63 cells using Annexin V-FITC\PI staining method. Statistically significant (*p* < 0.05) differences are shown by lowercase letters. a: (*p* < 0.05) with respect to the control untreated cells, b: (*p* < 0.05) with respect to PGLE group, and c: (*p* < 0.05) with respect to PdNPs group.

The probability of the PGLE, PdNPs, and PdNPs-PGLE inducing MG-63 cell apoptosis was tested using the Annexin V-FITC/PI dual labeling assay, and the obtained results were displayed in Figs. 11 and 12. Compared to control cells (Fig. 11a), the percentage of total apoptosis (early apoptosis and late apoptosis) in PGLE (Fig. 11b), PdNPs (Fig. 11c), and the PdNPs-PGLE (Fig. 11 d) treated MG-63 cells was significantly higher (*P* < 0.05). Moreover, the PdNPs-PGLE showed the highest % of total apoptosis compared to PdNPs and PGLE in MG-63 cells. Importantly, this finding is supported by additional molecular and cellular assays. Collectively, these findings support the interpretation that the PdNPs–PGLE formulation exerts a stronger apoptosis-associated cytotoxic effect in MG-63 cells than PGLE or PdNPs alone. This substantial elevation could be a result of the interaction between the phytoconstituents of *Psidium guajava* leaves extract and the palladium nanoparticles, leading to enhanced activation of apoptotic pathways. These findings are consistent with previous reports, including that of Gurunathan et al., who demonstrated that PdNPs synthesized using *Evolvulus alsinoides* leaf extract induced apoptosis in A2780 cells through caspase-3 activation and DNA fragmentation^83^.

#### Cell cycle assay

**Fig. 13:**
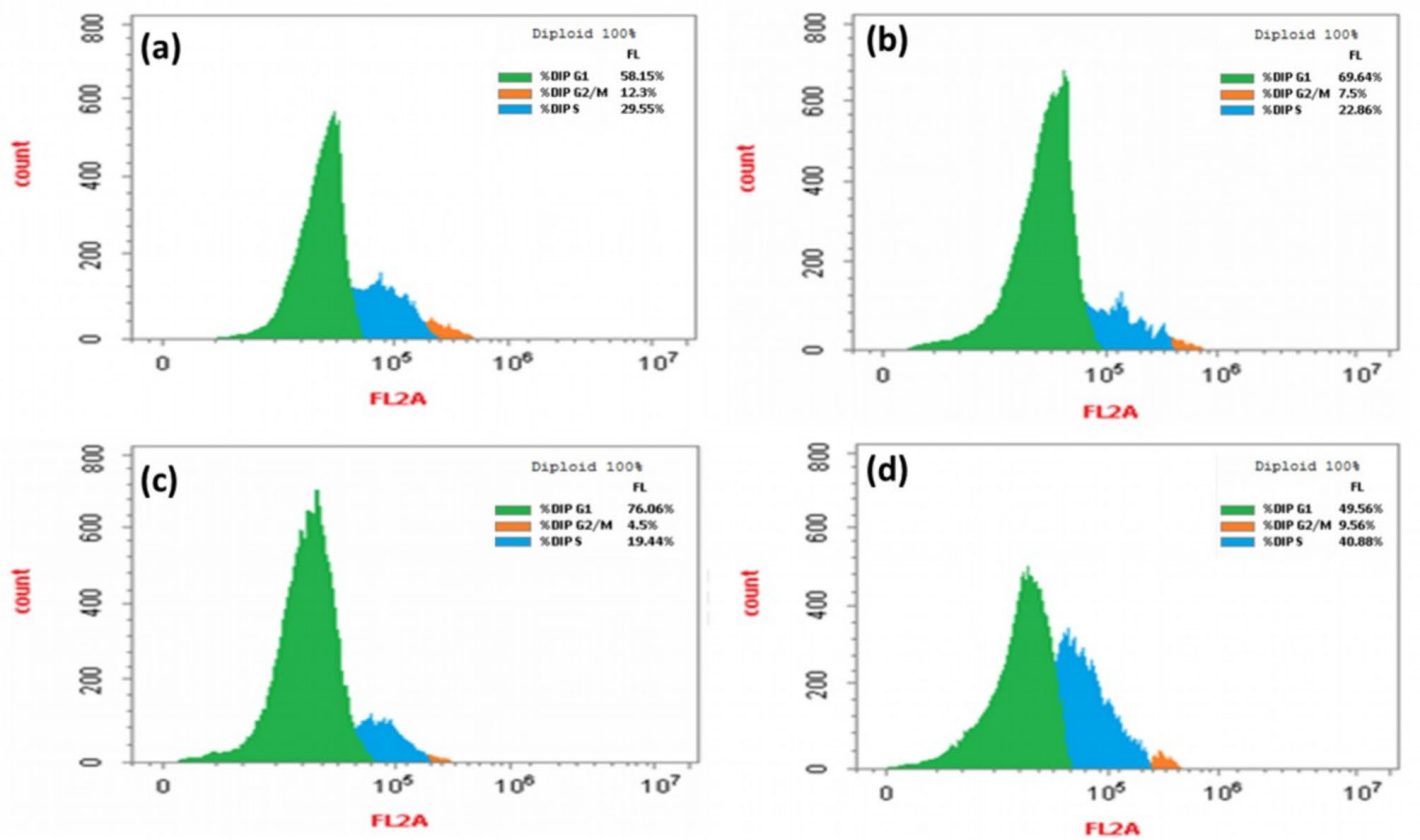
Cell cycle distribution at various stages in (a) control cells and MG-63 cells treated with (b) an IC_50_ dose of PGLE, (c) PdNPs, and (d) PdNPs-PGLE were analyzed using flow cytometry.

**Fig. 14:**
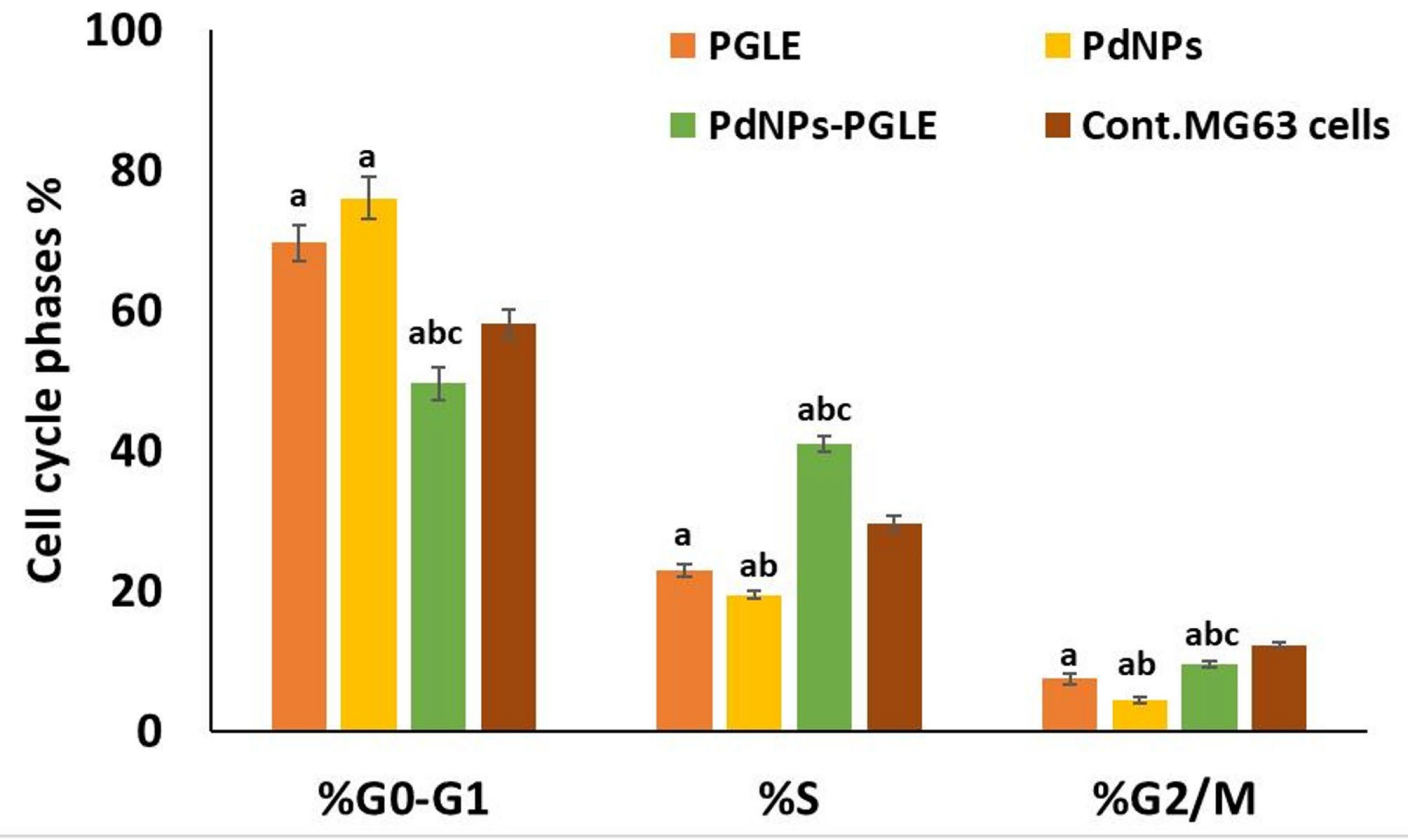
An illustration of a histogram that displays the percentage of cells in each stage of the cell cycle in untreated MG-63 cells and PGLE, PdNPs, and PdNPs-PGLE -treated cells. Statistically significant (*p* < 0.05) differences are shown by lowercase letters. a: (*p* < 0.05) with respect to the control untreated cells, b: (*p* < 0.05) with respect to PGLE group, and c: (*p* < 0.05) with respect to PdNPs group.

One of the main methods for stopping the spread of cancer is to stop the progression of the cell cycle^35^. Therefore, the ability to affect cell cycle phases was examined using flow cytometry. As seen in Fig. 13, the exposure of MG-63 to PGLE (Fig. 13b) and PdNPs (Fig. 13c) exhibited a significant increase of G0/G1 phase cells (69.64% and 76.06%, respectively) with (*p* < 0.05), a significant decrease of each S phase cell (22.86% and 19.44%, respectively), and a G2/M phase (7.5% and 4.5%, respectively) (*p* < 0.05) compared to control untreated cells (Fig. 13a). In contrast, the PdNPs-PGLE-treated cells (Fig. 13d) show a significant increase in the proportion of S phase (40.88%) (*p* < 0.05) and a significant decrease in the proportion of G0/G1 phase and G2/M phase cells (49.56%, and 9.56%) respectively (*p* < 0.05), compared to untreated cells. Also, the percentages of the cells in each phase of the cell cycle in the treated cells and control cells are represented in a representative histogram as shown in Fig. 14. Thus, cell cycle analysis showed that the biogenic PdNPs and PGLE blocked the MG-63 cell cycle, leading to G0/G1 phase arrest, which effectively inhibited cell proliferation, while the PdNPs-PGLE blocked the MG-63 cell cycle, leading to S phase arrest, which inhibited the DNA replication process, leading to inhibition of cell growth. This arrest not only limits proliferation but also sensitizes cells to apoptotic cell death, consistent with the observed increase in apoptotic population in treated MG-63 cells. The variations in the arrest phase observed in our study may therefore be attributed to differences in nanoparticle composition, cellular uptake, or the specific phytoconstituents of *Psidium guajava* involved in the bioreduction and capping processes. Overall, the cell cycle analysis provides compelling evidence that both PGLE and PdNPs suppress MG-63 cell proliferation by stimulating G0/G1 phase arrest, whereas the PdNPs–PGLE nanocomposite predominantly exerts its antiproliferative activity through S-phase arrest. These distinct mechanisms of cell cycle modulation contribute to the enhanced anticancer efficacy of the PdNPs–PGLE formulation and underline the potential of *Psidium guajava*–based nanomaterials as promising candidates for osteosarcoma therapy. Similarly, Gurunathan et al. demonstrated that the synthesized palladium nanoparticles using hesperidin showed an arrest in G0/G1 in ovarian cancer cells (SKOV3)- treated cells^84^.

## Clonogenic assay

**Fig. 15:**
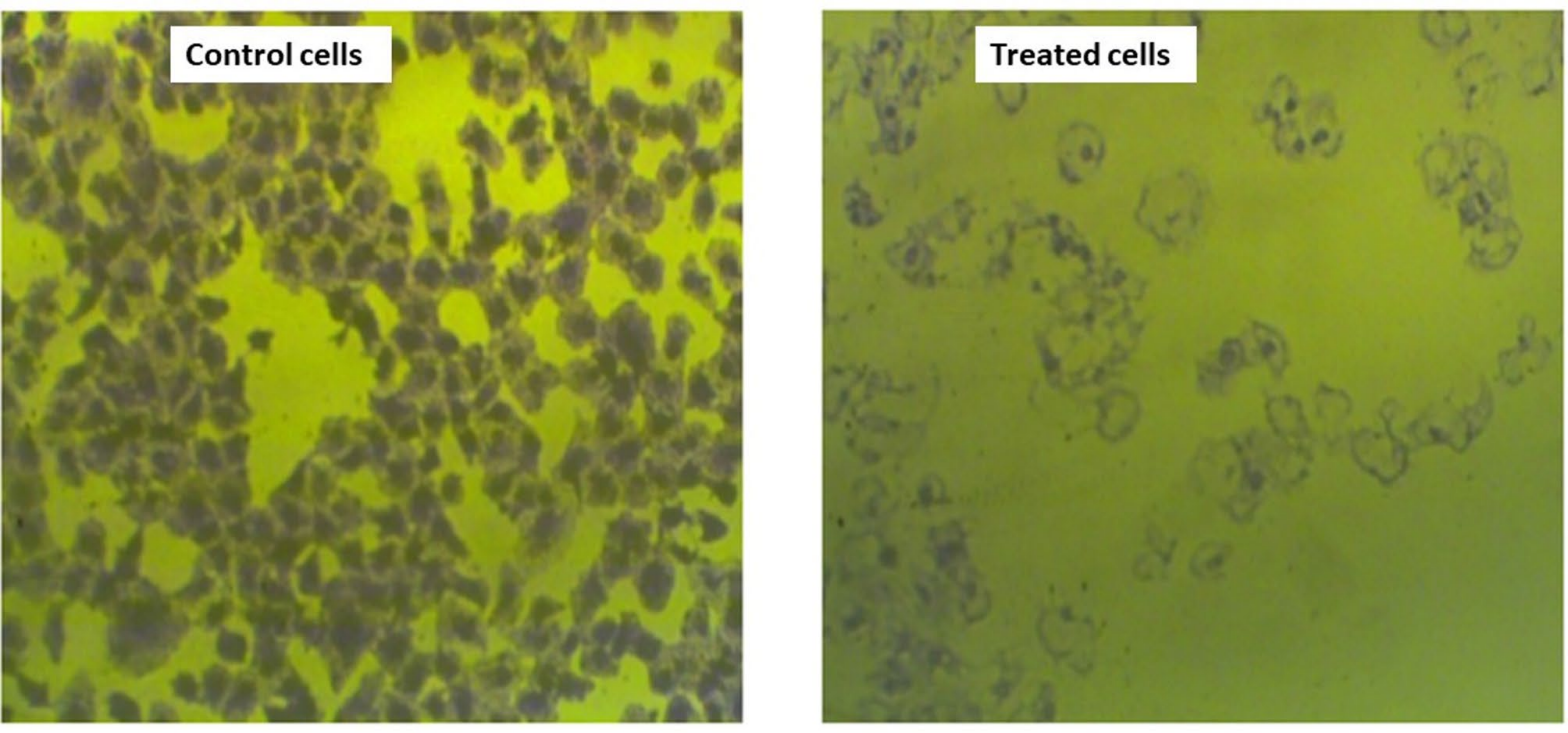
A microscopic evaluation of the effect of extract-suspended palladium nanoparticles (PdNPs-PGLE) on MG-63 cell survival. Control -untreated MG-63 cells create dense colonies, whereas cells treated with (PdNPs-PGLE) form significantly less colonies. This decline is due to the inhibitory action of (PdNPs-PGLE) on cell proliferation and viability.

This assay is an important measure of long-term cellular survival, as well as a single cell’s ability to divide and produce colonies indefinitely. Self-renewing mammalian cells produced from in vitro cultures and ex vivo tissue preparations are frequently discovered and quantified using this technique^85^. The results showed that extract-suspended palladium nanoparticles (PdNPs-PGLE) significantly decreased the colony-forming ability of MG-63 cells after 48 h of treatment. The reported plating efficiency (PE) was 0.9%, and the surviving fraction (SF) was 0.009 compared to the control untreated MG-63 cells, which had a plating efficiency of 100% and a surviving fraction of 1. Furthermore, stereomicroscopic assessment of plating efficiency and cell survival, (Fig. 15) demonstrated a significant reduction in both the density and number of MG-63 cells after treatment with PdNPs-PGLE compared to the untreated control group. Accordingly, these results suggest that PdNPs-PGLE have a strong anti-proliferative effect against the osteosarcoma cell line (MG-63). The near-complete suppression of colony formation indicates that PdNPs-PGLE have a significant anti-proliferative and cytostatic effect, severely affecting cancer cells’ reproductive viability even after treatment discontinuation.

## Cell scratch wound healing assay

**Fig. 16:**
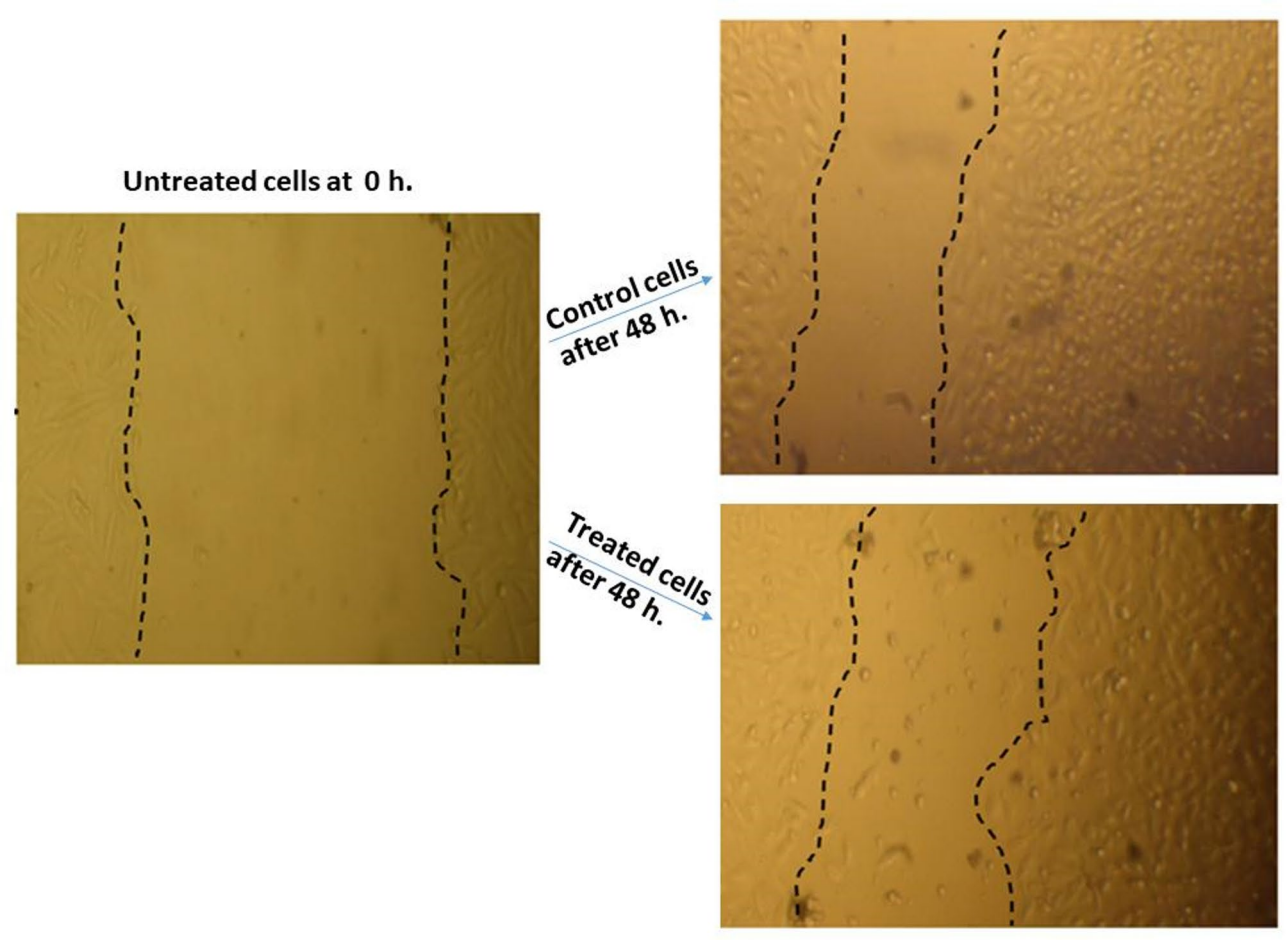
The migratory behavior of MG-63 cells was assessed over a 48-hour duration utilizing a wound healing assay observed via microscopy. Following the induction of a scratch in the MG-63 cell monolayer, the migration of both untreated control cells and cells treated with PdNPs-PGLE toward the wound site was evaluated at initial (0 h) and final (48 h) time points. The wound margins are delineated by dashed lines, and the extent of wound closure serves as an indicator of the cellular migratory response in each experimental condition.

. Cell migration is a crucial factor associated with tumor progression and metastatic potential^86^. In this investigation, a wound healing assay was used to determine if extract-suspended palladium nanoparticles (PdNPs-PGLE) might reduce the migratory rate of MG-63 cancer cells *in vitro*. Figure 16 shows that scratched MG-63 cells treated with PdNPs-PGLE had a higher wound closure percentage and migration rate (45.21% and 12.41 μm, respectively) than untreated control cells (58.78% and 16.14 μm). These data show that PdNPs-PGLE impaired the scratch healing capacity of the cells when compared to the control group, resulting in a lower wound closure percentage and migration rate in the treated cells. This observation shows that migratory behavior is reduced in vitro. Notably, this effect was accompanied by apoptosis-related alterations, which could explain the reduced scratch closure. Furthermore, the reduction in wound closure may be due in part to the concomitant S-phase arrest and apoptosis-related cytotoxicity observed in treated cells. As a result, while the current findings show an inhibitory effect of PdNPs-PGLE on *in vitro* wound healing, they do not provide conclusive evidence for an anti-metastatic mechanism. Additional research on cell migration and invasion is required to confirm this potential effect.

## Comet assay

**Fig. 17:**
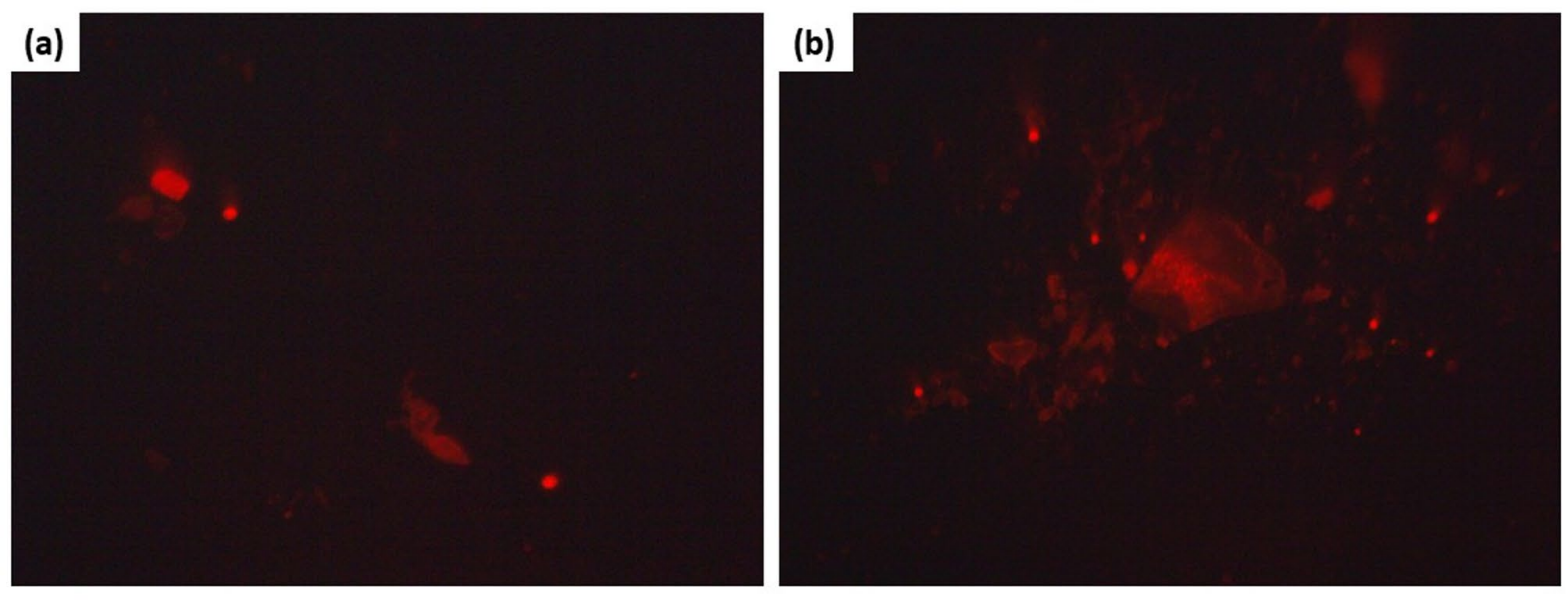
Fluorescence microscopy of the comet nucleus in (b) the PdNPs-PGLE treated MG-63 cells in comparison to an intact nucleus in (a) untreated MG-63 control cells.

The comet assay is widely used as a standard method for detecting DNA damage in genotoxicity tests and human biomonitoring studies. It is also a widely used method in ecotoxicology and environmental monitoring to research various animal and plant species^87^. The DNA damage that may result in the apoptosis-induced cells was studied by a comet assay. The PdNPs-PGLE-processed cells had an intense comet nucleus as a sign of DNA impairment, whereas the control cells had an intact nucleus, according to the fluorescence microscopic images in Fig. 17. As seen in Table 1, comet assay analysis revealed a significant increase in DNA damage in PdNPS-PGLE-treated MG-63 cells compared to control cells (P≺0.05), as evidenced by a marked rise in tail DNA content, and a decrease in head DNA.  These findings indicated that cells treated with PdNPs-PGLE were progressing toward apoptosis. According to the previous study of the green-synthesized silver nanoparticles’ effect on the genotoxicity of human lymphocytes, nanoparticles can generate reactive oxygen species (ROS), causing cell damage and oxidative stress. ROS generation and oxidative stress contribute to DNA damage, resulting in strand breaks^88^.

**Table 1:** The comet parameters in MG-63 cells processed with the PdNPs-PGLE and control untreated MG-63 cells. Data are presented as mean ± SD of three separate analyses. Statistically significant (*p* < 0.05) differences are shown by lowercase letters. a: (*p* < 0.05) with respect to the control MG-63 cells.

Control cells	Comet Length (µm)	Head Diameter (µm)	%DNA in Head	Tail Length (µm)	%DNA in Tail	Tail Moment	Grades
	11.34 ± 0.54	10.98 ± 0.54	99.8 ± 0.18	0.24 ± 0.21	0.19 ± 0.18	4.6 × 10⁻⁴ ± 7.9 × 10⁻⁴	0
Treated cells	27.72 ± 4.32^a^	13.68 ± 2.16	34.83 ± 5.49 ^a^	14.04 ± 2.16 ^a^	65.16 ± 5.49 ^a^	9.03 ± 0.63 ^a^	3

## ELISA assay

The apoptotic-related proteins (Bax, Bcl-2, cleaved caspase-3, and TP53), cell cycle-related proteins (CCNA and CDK2), and metastasis-related proteins (MMP-2 and MMP-9) were evaluated in the extract-suspended palladium nanoparticle-treated MG-63 cells to discover if the impact of PdNPs-PGLE on the apoptosis, migration, and cell cycle regulation was through affecting the regulation proteins or not. As displayed in Table 2, PdNPs-PGLE exhibited a significant upregulation in the expression level of Bax, TP53, and cleaved Caspase-3, followed by a significant downregulation of Bcl-2, CCNA, CDK2, MMP-2, and MMP-9, *p* < 0.001 in contrast to untreated control cells. According to the reports^89^, the Bcl-2 family is important in regulating apoptosis because its members operate as either antiapoptotic or proapoptotic controllers, such as Bcl-2 and Bax. Overexpression of Bax leads to mitochondrial permeability, which increases caspase-3 activation and triggers the production of cytochrome C into the cytoplasm^89^. As a result, activation of Caspase-3 causes impairment of DNA, apoptosis implementation, and a decrease in Bcl-2 gene expression levels. The tumor suppressor protein TP53 played a vital role in promoting apoptosis by inducing pro-apoptotic genes as the transcription factor implied upregulation, and this elevation in expression level was revealed from the apoptotic cell’s DNA damage recorded in the treated cells^90^. The current findings demonstrated a considerable rise in the expression levels of Bax, TP53, and cleaved caspase-3, as well as a significant reduction in Bcl-2 levels when contrasted with untreated cells (Table 2). These data suggest that the stimulation of apoptosis in PdNPs-PGLE-treated MG-63 cells happened mostly via the mitochondrial pathway.

One effective way to prevent tumor growth is to effectively block the cancer cell cycle progression^91^. The current results of cell cycle distribution analysis implied that the exposure of MG-63 cells to PdNPs-PGLE caused a significant accumulation of cells in S-phase, resulting in S-phase arrest. Cyclin-dependent kinase 2 (CDK2) and its associated complexes have been recognized as key regulators, playing a crucial role in the progression of the S phase in eukaryotic cells^91^. The downregulation of both CCNA and CDK2 might lead to the downregualtion of the CDK2/CCNA complex that associates with and phosphorylates several components of the DNA replication machinery^92^. Thus, the suppression of the activity of CDK2/CCNA complex could clarify the S phase arrest seen in this experiment. The current findings demonstrated that PdNPs-PGLE significantly decreased CDK2 and CCNA expression levels (Table 2), implying that the PdNPs-PGLE caused an arrest in S phase detected in the processed cells by inhibiting the CDK2 and CCNA complex activity.

One of the many phases in the invasion and spread of cancer is the breakdown of extracellular matrix (ECM). The metalloproteinases in the matrix (MMPs), such as MMP-2 and MMP-9, were a class of proteinases that can catalyze the cleavage of extracellular matrix components, promoting migration, invasion, and metastasis of tumor cells^93,94^. Furthermore, it is regarded as the initial critical way to stop the spread of cancer cells is the reduction of MMP expression levels^95^. To establish whether the reported antimetastatic impact in the PdNPs-PGLE-treated cells was related to a decrease in MMP expression, the effect of the PdNPs-PGLE on expression levels of MMP-9 and MMP-2 proteins in treated cells was examined. As shown in Table 2, the PdNPs-PGLE markedly reduced the expression levels of MMP-9 and MMP-2 in comparison to control cells, indicating its potential to inhibit MG-63 cell migration by lowering these protein levels.

**Table 2:** The effect of the IC_50_ dose of the PdNPs-PGLE on the protein levels of cleaved caspase-3, TP53, Bax, CDK2, CCNA, Bcl-2, MMP-2, and MMP-9 in MG-63 treated cells contrasting to the protein levels of the control cells, utilizing the ELISA method. Data are presented as mean ± SD of three separate analyses. Statistically significant (*p* < 0.05) differences are shown by lowercase letters. a: (*p* < 0.05) with respect to the control MG-63 cells.

Protein	Control cells (mean ± SD) (pg/ml)	Treated cells (mean ± SD) (pg/ml)
Bax	108.33 ± 4.2	a 180.03 ± 6.9
Bcl-2	17,050 ± 580	a 7130 ± 240
cleaved Caspase-3	48.98 ± 1.9	a 124 ± 4.8
TP53	176.95 ± 6.68	a 483.58 ± 18.24
CDK2	8150 ± 270	a 4740 ± 160
CCNA	3040 ± 120	a 1800 ± 70
MMP-2	194,630 ± 7450	a 137,840 ± 5300
MMP-9	2443.55 ± 94.9	a 1644.08 ± 63.8

## Antioxidant activity using DPPH Assay

**Fig. 18:**
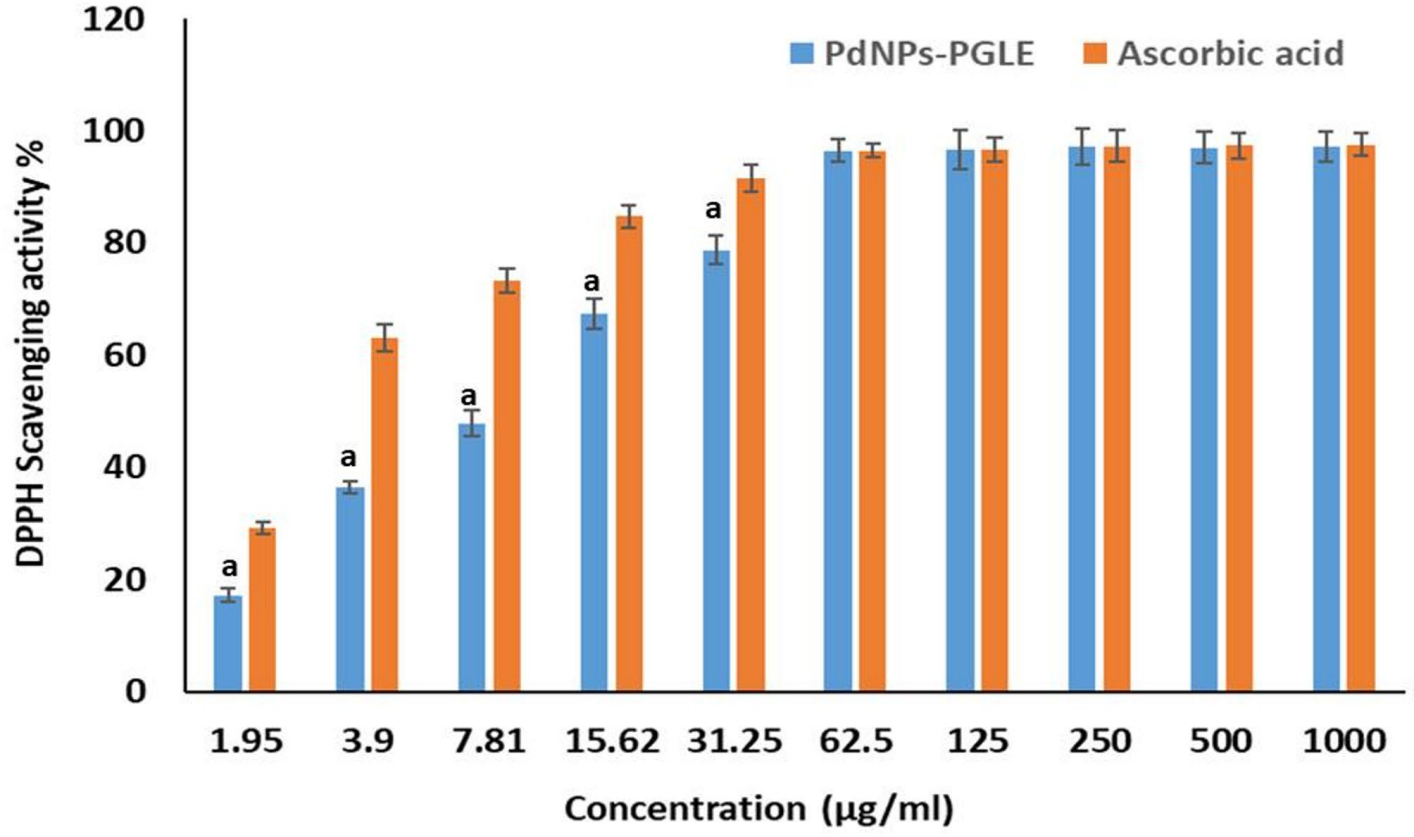
The investigated antioxidant properties of PdNPs-PGLE in comparison to vitamin C against DPPH radicals were assessed at different dosages. Data are presented as mean ± SD of three separate analyses. Statistically significant (*p* < 0.05) differences are shown by lowercase letters. a: (*p* < 0.05) with respect to ascorbic acid.

The DPPH assay is a simple, rapid, and inexpensive assay that measures the scavenging capacity of antioxidants against the free radical DPPH^96^. DPPH, a stable free radical, undergoes reduction upon accepting electrons and protons from a donor, such as nanoparticles, resulting in the formation of the stable yellowish DPPH-H molecule^97^. As revealed in Fig. 18, the free radical-scavenging capacity of extract-suspended palladium nanoparticles against 2,2-diphenyl-1-picrylhydrazyl (DPPH) radicals was evaluated. Various concentrations of extract-suspended palladium nanoparticles (3.9, 7.8, 15.62, 31.25, 62.5, 125, 250, 500, and 1000 µg/mL) were tested, with ascorbic acid serving as a standard reference. The percentage of DPPH radical scavenging exhibited a positive correlation with increasing concentration, ranging from 3.9 to 1000 µg/mL. At the highest concentration tested (1000 µg/mL), the extract-suspended palladium nanoparticles achieved a maximum DPPH scavenging activity of 97.14%, with an IC_50_ value of 8.89 ± 0.13 µg/mL. In comparison, ascorbic acid demonstrated a scavenging activity of 97.59% at the same concentration (1000 µg/mL) and an IC_50_ value of 3.97 ± 0.03 µg/mL. As shown in Fig. 18, the concentration from 62.5 µg/mL to 1000 µg/mL exhibited the same DPPH scavenging activity. The findings are consistent with previous studies reporting the antioxidant properties of PdNPs synthesized using various plant extracts, including Anogeissus latifolia^98^, Urtica^82^, Agaricus bisporus^75^, and Saussurea costus^99^.

## Conclusion

This study illustrates the effective green production of palladium nanoparticles (PdNPs) through the use of the aqueous leaf extract from *Psidium guajava*, thereby establishing an environmentally friendly, economical, and sustainable method for the synthesis of nanoparticles. The biosynthesized PdNPs exhibited uniform spherical morphology with nanoscale dimensions and high colloidal stability, confirming the efficiency of the phytochemical-mediated synthesis process. Biological evaluations revealed that the *Psidium guajava* leaf extract–suspended palladium nanoparticles (PdNPs–PGLE) exerted pronounced cytotoxicity against MG-63 human osteosarcoma cells, surpassing the effects of the extract or nanoparticles alone. The increased anticancer activity was linked to several mechanisms, such as the upregulation of Bax, Caspase-3, and TP53 proteins, which led to apoptosis, and the downregulation of anti-apoptotic and pro-metastatic proteins like Bcl-2, MMP-2, and MMP-9. Moreover, PdNPs–PGLE treatment was associated with S-phase cell cycle arrest, reduced clonogenic survival, and decreased cell migration, suggesting antiproliferative activity and a possible inhibitory effect on cell motility. The *Psidium guajava*–mediated PdNPs could potentially serve as a nanotherapeutic candidate for osteosarcoma, possibly attributable to the combined bioactivity of the phytoconstituents and the metallic core; however, in *vivo* investigations and molecular pathway studies are warranted to elucidate their clinical relevance and therapeutic safety profile.

## Data Availability

The datasets used and/or analysed during the current study available from the corresponding author on reasonable request.
